# Adult attachment, loneliness, and self-reported sleep outcomes: A secondary analysis of the Touch Test Study

**DOI:** 10.1371/journal.pone.0356812

**Published:** 2026-09-08

**Authors:** Jolana Samii, Aikaterini Vafeiadou, Angela Rowe, Christopher Kent, Michael J. Banissy

**Affiliations:** 1 School of Psychological Science, University of Bristol, Bristol, United Kingdom; 2 Department of Psychology, Goldsmiths, University of London, London, United Kingdom; Hangzhou Normal University, CHINA

## Abstract

**Objective:**

Attachment dimensions and loneliness have independently been associated with sleep outcomes, with lower attachment insecurity and lower loneliness generally related to better sleep. However, it remains unclear whether attachment dimensions and loneliness interact in relation to sleep. The current study aimed to examine whether attachment anxiety and attachment avoidance moderated associations between loneliness and self-reported sleep outcomes.

**Design and measures:**

A secondary data analysis was conducted using data from the Touch Test Study, which was originally run to measure outcomes and attitudes towards touch. Attachment dimensions, loneliness, and the interactions between loneliness and each attachment dimension were treated as independent variables. Sleep duration, sleep quality, time to fall asleep, and wake after sleep onset were treated as dependent variables. Adult participants from the UK (N = 15,049) with no health conditions or impairments were included in the study, having been originally recruited through opportunity sampling. The final analytic sample for the main models ranged from N = 11,901 to N = 11,916 across sleep outcomes.

**Results:**

Ordinal logistic regression showed that individuals with higher loneliness reported shorter sleep duration at higher levels of avoidant attachment. This was the only significant attachment × loneliness interaction in the adjusted models and remained significant after correction for multiple testing and in a sensitivity analysis excluding participants who reported sleeping more than nine hours.

**Conclusion:**

These findings provide limited evidence that loneliness and avoidant attachment interact in relation to self-reported sleep duration. However, attachment × loneliness interactions were not observed for sleep quality, sleep onset, or wake after sleep onset. The findings suggest that attachment and loneliness may be relevant relational factors in sleep research, while recognising that the moderation effect observed here was specific to sleep duration and small in magnitude.

## 1. Introduction

The consequences of poor sleep are extensively documented, encompassing physical, cognitive, and mental health implications that can impact various aspects of everyday life [1–3]. For example, poor sleep quality has been associated with depression and depressive symptoms in college students, in addition to increased occupational stress [2,3]. With this in mind, understanding factors contributing to suboptimal sleep is crucial in addressing this widespread issue.

Much work has linked sleep quality to various aspects of daytime functioning, including an individual’s attitudes and traits [4] and their social relationships [5–7]. Interpersonal relationships play a vital role in both mental and physical health and the mechanisms by which they impact health are now an important focus in psychology research [8]. Both social connection and disconnection appear to influence our health behaviours and physiological responses closely related to our health. Pietromonaco and Collins [8] suggest a need for research on contextual factors and individual differences that may be affecting our health in the context of interpersonal psychology. Both the perception of the availability and quality of our interpersonal relationships may be important in predicting sleep quality [5].

In terms of the perception of the availability of interpersonal relationships, loneliness may play an important role in our sleep quality, though many of the mechanisms by which loneliness may affect sleep are still unknown [9]. Loneliness may be defined as an emotional response to a discrepancy between actual levels of social contact and support and desired levels of social contact and support [10]. Individuals with higher levels of loneliness tend to report lower sleep quality in addition to increased stress, with consequent lower sleep quality for their partners [11]. Based on the findings, the stress resulting from feelings of loneliness may negatively impact individuals’ sleep.

The quality of important interpersonal relationships, in addition to their perceived availability, would appear also to be important when it comes to sleep. One factor reported to impact sleep outcomes throughout the life course, and that appears to be associated with loneliness, is attachment style [12,13]. Attachment theory has become an important theory in health psychology. It is now acknowledged that our childhood and later attachment experiences become internalised in mental models that can affect our health and behaviour in later life, including how we cope with stress and illness [12,14,15]. Adult attachment style affects behaviour, emotions and cognition in relationships and other aspects of social life [16,17]. There has been an increased tendency to use attachment dimensions to measure adult attachment, where individuals are studied based on their level of anxious and avoidant attachment as opposed to being placed in 3 or 4 discrete categories or styles of attachment [18,19]. This dimensional approach is the one we take in the current study. Below, we outline the attachment dimensions before also explaining how these map onto a categorical model of attachment styles, as several of the relevant studies on sleep and attachment we review here have used such measures of attachment style. Individuals high on the anxious attachment dimension tend to feel insecure in a relationship and fear abandonment. Consequently, anxiously attached individuals can crave more attention and emotional support from their partner, as a form of reassurance, than they receive. On the other hand, those high on the avoidant dimensions tend to move away from intimacy and closeness and are likely to withdraw when faced with relationship challenges [18,19]. Furthermore, high attachment anxious individuals use hyperactivating strategies during stressful situations, in which their behaviours and emotions become heightened to elicit more attention [20], high avoidant individuals tend towards deactivating strategies where attachment-related threats and attachment needs are downplayed [20]. The intersections of the two dimensions would correspond to the preoccupied (high anxious, low avoidance) and dismissing avoidant (high avoidant, low anxious) attachment styles in categorical models of attachment [16,21], while individuals characterised by low levels of both anxiety and avoidance are considered ‘secure’ and more likely to be comfortable with intimacy and dependence and to view their partnership in a more positive light [18,19]. If an individual is high on both dimensions, this would correspond to fearful-avoidance, which is characterised by the use of both hyperactivating and deactivating strategies [20,22]. Attachment styles have been found to be related to health as they are involved in affect regulation, which in turn is related to how stress is managed and behaviour is regulated, including health-related behaviours. In this way, attachment styles/dimensions influence health-related behaviours and impact general mental and physical health outcomes [23].

A comprehensive investigation of the nexus between attachment styles and sleep outcomes throughout the life course by Adams et al. [12] revealed noteworthy insights. During adulthood, an anxious or avoidant attachment style emerged as a salient correlate of insufficient sleep [12]. Furthermore, individuals high in attachment anxiety experience greater sleep decline while separated from their partners or when experiencing attachment threats such as situations in which they feel rejected by their partners [12]. An avoidant attachment style also predicts poor sleep for individuals not in relationships [24]. A meta-analysis conducted by Hu and colleagues [6] also showed that there appears to be a correlation of a small effect size between insecure attachment and the sleep of an individual and their partner. Insecure attachment, when an individual has higher scores of anxious attachment or avoidant attachment, appears to negatively affect different measures of sleep, including self-reported sleep quality, sleep latency, wake-after-sleep onset, and daytime sleepiness. Previous research suggested that an anxious attachment style, but not avoidant, may be associated with sleep disturbances [25]. The study by Carmichael and Reis [25] showed that anxious attachment style predicted diminished sleep quality even when controlling for symptoms of depression or general anxious tendencies, thereby underscoring the specificity of the relationship between attachment style and sleep outcomes.

Importantly for the purposes of the current research, studies show that a secure attachment style has been linked to lower levels of loneliness [13]. By contrast, insecure attachment styles appear to be linked to higher levels of loneliness [26]. One mechanism by which loneliness and attachment style may be linked is through maladaptive schema, which directs the responses of the individual to their attachment models [27]. Individuals with avoidant and anxious attachment styles tend to view their relationships through a schema of rejection-disconnection where they react to their attachment figure through distrust, instability, emotional deprivation, shame, abandonment and rejection. In addition to influencing the individual’s attachment responses and behaviour, such a schema may lead to increased loneliness [27]. Furthermore, anxiously attached individuals may feel lonelier due to a combination of wanting to gain people’s acceptance and feeling higher levels of rejection anxiety, while avoidant individuals may feel lonely due to a mistrust of other individuals in addition to a generally negative view of human nature [28].

Attachment and loneliness may be linked to sleep through psychological and behavioural pathways. Attachment insecurity is associated with difficulties in emotion regulation, heightened sensitivity to relational threat, and dimension-related differences in responses to interpersonal stress [29,30]. Loneliness, in turn, reflects perceived social disconnection and has been linked to heightened vigilance for social threat and poorer sleep [9,31]. Sleep may therefore be disrupted when individuals feel socially disconnected, particularly if their attachment orientation makes relational threat or interpersonal distance more difficult to regulate. In this sense, loneliness may not be associated with sleep uniformly across individuals; rather, its association with sleep may depend partly on attachment-related individual differences. This provides the rationale for testing whether attachment anxiety and attachment avoidance moderate associations between loneliness and self-reported sleep outcomes. Furthermore, a tendency towards maladaptive schema used by individuals with insecure attachment, which might heighten feelings of loneliness, may also lead to decreased sleep quality [27].

Existing literature has extensively explored the individual associations between loneliness and sleep and between attachment and sleep. However, a gap in the literature exists in exploring potential interactive dynamics between attachment dimensions and loneliness, particularly in their collective association with sleep outcomes. Research appears to suggest psychological and biological factors that link loneliness, attachment, and sleep together. Still, there is a lack of studies directly examining these factors together. Therefore, the present study investigates how insecure attachment might moderate the relationship between loneliness and sleep patterns (sleep duration, perceived sleep quality, latency to sleep onset, and the temporal duration participants remained awake after nocturnal awakenings). By examining the interrelationships between attachment dimensions, loneliness, and sleep parameters, this research extends the literature on psychological factors that appear linked to sleep patterns to include relational factors. Specifically, we tested the following hypotheses:

### Hypotheses

1. There will be a positive and significant correlation between higher traits of each insecure attachment dimension (anxious and avoidant) and loneliness.
2. Higher levels of each insecure attachment dimension (anxious and avoidant) will significantly moderate the relationship between loneliness and sleep (sleep quality, sleep duration, sleep onset, and wake-after-sleep onset) where higher scores of loneliness will be associated with shorter sleep duration, lower sleep quality, longer sleep onset, and longer wake-after-sleep onset at higher scores of each insecure attachment dimension.

## 2. Method

### 2.1. Data collection

The data were drawn from a large worldwide survey, conducted in collaboration with the British Broadcasting Corporation and Wellcome Collection, completed between 21st January and 30th March 2020. The dataset can be accessed at: https://reshare.ukdataservice.ac.uk/854471. For this study, the data was accessed on the 16th of October 2023.

The original study explored touch attitudes and touch in relation to health and psychological outcomes [32,33]. In that study, participation was online and voluntary. Participants were given an online link through radio broadcasts and associated websites. Participants gave informed written consent online before completing the questionnaire and were not compensated for participation. For a full list of measures included in the survey, please see the attached document in the following linked registration: https://osf.io/2xgny. Only certain blocks from the original questionnaire were included in the secondary analysis: loneliness, attachment style, demographics, and sleep. The Touch Test used validated measures of sleep, attachment style, and loneliness (see Measures section). Ethical approval from the local university was given for the original survey. Ethical approval for the current secondary analysis was provided by the University of Bristol School of Psychological Science Research Ethics Committee on the 3rd of November 2023. The ethics approval code was 16127. The study was pre-registered on October 10th 2023 prior to data analysis (https://archive.org/details/osf-registrations-5qm8n-v1). A deviation from the preregistered analysis plan included removal of the three-way interaction between attachment anxiety, attachment avoidance, and loneliness because the revised analysis focused on the two theoretically specified attachment × loneliness interactions.

### 2.2. Study participants

Participants were all 18 years or older. For the current study, inclusion criteria included being a man or woman, not having any psychological or physical health condition, and being from the UK. These selection criteria were chosen to ensure maximum sample size and statistical power from the data set as the representation from other groups (non-UK, selected gender as ‘other’) was substantially lower, with some countries only having one responder (e.g., the USA only represented 1% of the sample with 763 participants). The sample consisted of 15049 participants. Participation information for each analysis is mentioned in the results section.

#### 2.2.1. Participant characteristics.

The 15049 participants included in the analyses were 75.4% women (*N* = 11 343) compared to 24.6% men (*N* = 3706). The number of participants included in each analysis is mentioned in the results section. Participants were aged 18–92, with a mean age of 56.3 (*SD* = 13.79). Most participants were of a white ethnicity (95.8%, *N* = 14417). For full participant characteristics, participation exclusion, and excluded participants with missing data, see the supplemental methods section.

### 2.3. Measures

Attachment Dimensions: Attachment dimensions (attachment anxiety and avoidance) were measured with the 12-item Experiences in Close Relationships Questionnaire (ECR-12) [34]. The questionnaire has two subscales that indicate the individual’s anxious or avoidant attachment dimension level, with higher scores indicating higher levels of each attachment dimension. Answers are ranked on a 7-point scale and range from ‘strongly disagree’ to ‘strongly agree’. Examples include ‘it helps to turn to my partner in times of need’ (avoidant dimension) and ‘I need a lot of reassurance that I am loved by my partner’ (anxious dimension). Convergent validity with relationship satisfaction scales and psychological distress has been assessed as adequate, with good internal consistency for both subscales (Cronbach’s alpha between.74 and.82 for avoidance and.78 to.87 for anxiety) [34]. Attachment anxiety and attachment avoidance scores were calculated separately by averaging valid responses within each six-item subscale and multiplying the mean by six. This produced subscale scores ranging from 6 to 42, with higher scores indicating higher attachment anxiety or attachment avoidance, respectively. In the current analytic sample, internal consistency was good for both the anxious attachment subscale, α = .85, and the avoidant attachment subscale, α = .87.

Loneliness: To measure loneliness, the UCLA loneliness scale 4-item version (UCLA-LS) was used [35]. The measure consists of a shorter version of the original 20-item scale and is rated on a 4-point scale with answers ranging from ‘never’ to ‘always’. An example question is ‘How often do you feel that you are “in tune” with the people around you?’. Two of the items in the scale are reverse-scored. Higher total scores indicate higher loneliness. Total loneliness scores ranged from 4 to 16, with higher scores indicating greater loneliness. The UCLA-LS is a valid and reliable measure of loneliness with a good internal consistency (α = 0.94) and concurrent validity compared to depression and anxiety scales [35]. Panayiotou et al. [36] identified this 4-item version of the UCLA loneliness scale as a robust and reliable measure of loneliness across adult age groups. In the current analytic sample, internal consistency for the four-item loneliness measure was acceptable (α = .73).

Sleep: Sleep variables were adapted from the Pittsburgh Sleep Quality Index (PSQI) [37,38]. Sleep duration was measured by asking the participants how many hours they had slept on average per night in the past month, with answers being ‘less than 5 hours’, ‘5-6 hours’, ‘6-7 hours’, ‘7-8 hours’, ‘8-9 hours’, and ‘more than 9 hours’. Sleep quality was measured using the item ‘During the past month, how would you rate your sleep quality overall?’ with answers fitting a 4-point scale from ‘very bad’ to ‘very good’. Sleep latency and wake after sleep onset answers ranged from ‘0–15 min’ to ‘61 min or more’ and measured how long it took participants to fall asleep and how long they stayed awake if they woke up at night. As these sleep outcomes were selected items adapted from the PSQI rather than the full PSQI scale, they were analysed as separate self-reported sleep outcomes rather than as a composite PSQI score.

### 2.4. Analysis

All analyses were conducted using SPSS and R. Preliminary analyses examined the correlations between attachment dimensions and loneliness. Gender differences in loneliness and attachment dimensions were examined using independent-samples t-tests, and correlations were run to examine whether age and participation end date were associated with attachment dimensions, loneliness, and sleep variables. Age and gender were included as covariates because both have been associated with sleep and psychosocial outcomes in prior research. Participation end date was included to account for potential timing effects in the survey, as previous analyses of the Touch Test dataset found associations between participation end date and sleep variables [38], and data collection overlapped with the early stages of the COVID-19 pandemic.

To examine the associations between loneliness, attachment dimensions, and sleep, four separate ordinal logistic regression models were run, with sleep duration, sleep quality, sleep onset, and wake after sleep onset entered as dependent variables. The same adjusted model was used for each sleep outcome. Predictors were avoidant attachment, anxious attachment, loneliness, the interaction between avoidant attachment and loneliness, the interaction between anxious attachment and loneliness, age, gender, and participation end date. Main effects were mean-centred before creating the interaction terms. Only participants who completed all loneliness and attachment items and had available data for the relevant sleep outcome were included in each model; missing data were handled using complete-case analysis. The final analytic sample was *N* = 11,914 for sleep duration, *N* = 11,916 for sleep quality, *N* = 11,908 for sleep onset, and *N* = 11,901 for wake after sleep onset. The primary focus was on the two attachment x loneliness interaction terms across the four sleep outcomes, resulting in eight primary interaction tests. Benjamini-Hochberg false discovery rate correction was applied across these eight interaction tests. Multicollinearity was assessed using variance inflation factors (VIFs), which were all below 1.40. Because very long sleep may not represent better sleep health, and because the sleep-duration model treated sleep duration as an ordered outcome, a sensitivity analysis was conducted excluding participants who reported sleeping more than nine hours per night. Missing data were handled using complete-case analysis for each model. Participants were included in a given ordinal logistic regression model if they had complete data for the relevant sleep outcome, attachment anxiety, attachment avoidance, loneliness, age, gender, and participation end date. The number of participants included therefore differed slightly across sleep outcomes. A participant flow table is provided to show the number of participants excluded at each stage and the final analytic sample for each model. Model diagnostics, including model fit statistics and tests of the proportional odds assumption, were examined for all four ordinal logistic regression models and are reported in the Supplementary Material. The following formula was used for the ordinal logistic regression:


$$\begin{array}{c} \begin{array}{c} \text{Sleep outcome }=\text{ centred avoidance }+\text{ centred anxiety }+\text{ centred loneliness }+\text{ centred avoidance }\\ \times \text{ centred loneliness }+\text{ centred anxiety }\times \text{ centred loneliness }+\text{ age }+\text{ gender }+\text{ participation end date} \end{array} \end{array}$$

### 2.5. Power analysis

We utilized the InteractionPoweR() function in R to estimate the sample size required for detecting interaction effects in ordinal outcomes [39]. This function integrates population level Pearson’s r, which quantifies the strength of the relationship between interacting variables and the outcome. For the main effects, we selected r coefficient effect sizes in alignment with findings from the literature on attachment and loneliness where correlation values appear to be around r = .45 for both types of insecure attachment [40]. In addition, it is generally recognized that the r coefficients for interaction effects are usually small [39]. In light of this, our analysis examined how statistical power varied Pearson’s r values (r = 0.05 to 0.1), assessing the interplay between variables x1 and x2 and their combined effect on the outcome variable (y).

Accordingly, we performed four separate power analyses corresponding to each of our outcomes of interest (y): sleep duration, sleep quality, wake after sleep onset (WASO), and sleep latency. Each analysis was designed to determine the association of the interaction between anxious attachment (x1) and loneliness (x2) on these specific sleep-related variables.

For each sleep variable, a minimum sample size of *N* = 2500 was required to detect a small interaction effect (r = 0.05) with 80% power. For larger r values (r > 0.05), the power reached 100% for sample sizes of *N* > 6000. As a healthy UK sample was the only sample with over 2500 participants, only the UK sample was used in this study to reach the minimum power threshold. Because the present study used a large secondary dataset, the power analysis should be interpreted as a guide to the detectable interaction effect size rather than as a prospective design-based sample size calculation.

## 3. Results

### 3.1. Preliminary analysis

#### 3.1.1. Age, participation end date, and gender as covariates.

Preliminary analyses revealed mean differences in gender with loneliness (Cohen’s d = 0.315, p < .05). Age was significantly negatively correlated with anxious attachment (r = −.13, p < .05) and significantly positively correlated with avoidant attachment (r = .06, p < .05). Participation end date was correlated with anxious attachment (r = −.019) and avoidant attachment (r = .019, p < .05). Age, gender, and participation end date were retained as covariates in all models based on their theoretical and methodological relevance to sleep and the timing of data collection.

#### 3.1.2. Loneliness and attachment.

As loneliness and both attachment dimensions were not normally distributed (*p* < .05), Spearman’s rank correlation was used to assess the association between loneliness and attachment dimensions.

There was a significant positive correlation between loneliness and the avoidant attachment dimension (*r* (11989)=.42, *p* < .01) with 17.64% of the variance in loneliness accounted for by the avoidant attachment dimension. There was a significant positive correlation between loneliness and the anxious attachment dimension (*r* (12001) =.36, *p* < .01), with 12.96% of the variance in loneliness accounted for by the anxious attachment dimension.

There was also a significant positive correlation between the anxious and avoidant attachment dimensions (*r* (12087) =.11, *p* < .01), with 1.21% of the variance in the avoidant attachment dimension accounted for by the anxious attachment dimension.

### 3.2. Main analysis

Four ordinal logistic regression models were run to examine the association between the independent variables (avoidant attachment, anxious attachment, loneliness, loneliness x avoidant attachment, and loneliness x anxious attachment) and the dependent sleep variables (sleep duration, sleep quality, sleep onset, and wake-after-sleep onset). Age, gender, and participation end date were added into the model as covariates. The main effects were centered prior to creating the interaction terms. There were 11,914 participants in the model for sleep duration, 11,916 in the model for sleep quality, 11,908 in the model for sleep onset, and 11,901 in the model for wake-after-sleep onset. The eight attachment x loneliness interaction tests were corrected using Benjamini-Hochberg false discovery rate correction. Only the avoidant attachment x loneliness interaction for sleep duration remained significant after correction (FDR-adjusted p = .020).

**Fig 1:**
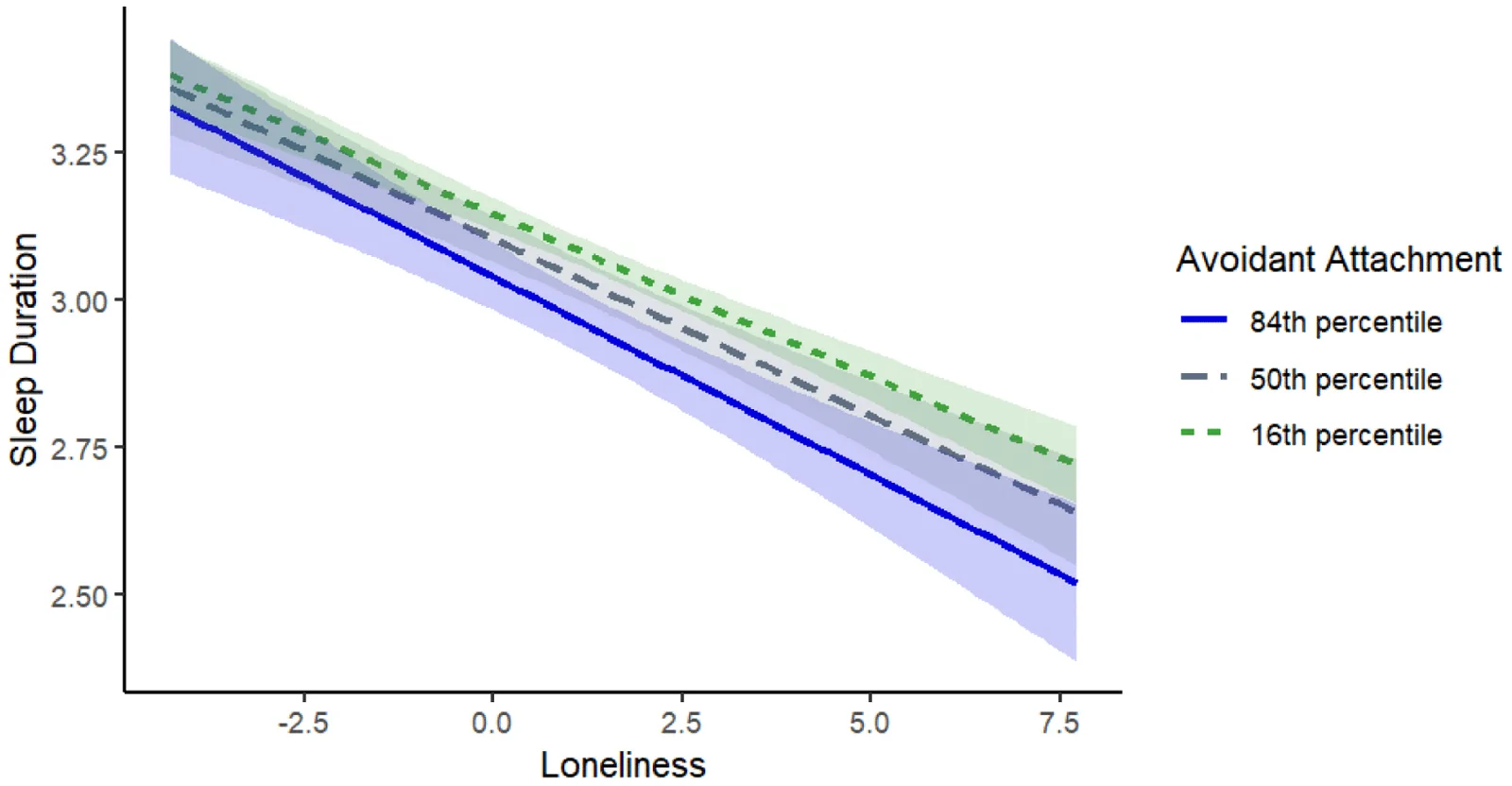
Moderation of centered avoidant attachment of the relationship between centered loneliness and sleep duration while controlling for age, gender, participation end date, and anxious attachment. Higher values on the sleep-duration axis indicate longer sleep duration; higher loneliness was associated with shorter sleep duration, particularly at higher levels of avoidant attachment.

Higher avoidant attachment was significantly associated with shorter sleep duration (OR =.990, 95% CI [.985,.995], B = −.010, p < .001). Higher anxious attachment was also significantly associated with shorter sleep duration (OR =.987, 95% CI [.983,.991], B = −.013, p < .001). Higher loneliness was associated with shorter sleep duration (OR =.924, 95% CI [.909,.939], B = −.079, p < .001). There was a significant interaction between avoidant attachment and loneliness in predicting sleep duration (OR =.997, 95% CI [.996,.999], B = −.003, p = .002; FDR-adjusted p = .020) (see Table 1). The interaction between anxious attachment and loneliness was not significant. Simple slopes analysis indicated that loneliness was significantly associated with shorter sleep duration at higher levels of avoidant attachment but not at lower levels of avoidant attachment (see Table 2). The interaction between avoidant attachment and loneliness was represented in an interaction plot using a customised R script (see Fig 1). A sensitivity analysis excluding participants who reported sleeping more than nine hours showed the same pattern of results for the avoidant attachment x loneliness interaction (OR =.997, 95% CI [.995,.999], B = −.003, p = .002). The sleep-duration model provided a significantly better fit than the intercept-only model, χ^2^(8) = 169.45, p < .001, with a small pseudo-R^2^ value (Nagelkerke R^2^ = .019). Pearson and deviance goodness-of-fit tests did not indicate poor model fit, p = .066 and p = 1.000, respectively. However, the test of parallel lines was significant, χ^2^(24) = 109.31, p < .001, suggesting that the proportional odds assumption may have been violated. Therefore, the sleep-duration findings should be interpreted cautiously. However, we retained the ordinal specification because it reflects the original ordered response categories and because sensitivity analyses yielded substantively similar conclusions.

**Table 1:** Predictor effects on sleep duration.

Predictor	OR [95% CI]	*p*	B
Age	.990 [.987–.992]	<.001*	−.010
Gender (Men)	1.023 [.947–1.106]	.562	.023
women	1 ref.	ref.	ref.
Participation End Date	1.001 [.999–1.003]	.270	.001
Avoidant Attachment	.990 [.985–.995]	<.001*	−.010
Anxious Attachment	.987 [.983–.991]	<.001*	−.013
Loneliness	.924 [.909–.939]	<.001*	−.079
Loneliness x Avoidant Attachment	.997 [.996–.999]	.002*	−.003
Loneliness x Anxious Attachment	1.000 [.998–1.002]	.974	.000

**Table 2:** Simple slopes analysis for the moderation of avoidant attachment of the relationship between loneliness and sleep duration.

Model	*OR* [95% CI]	*p*	*B*
Loneliness when: Low Avoidance X Loneliness, Low Avoidance, Loneliness	.999 [.956–1,043]	.950	−.001
Loneliness when: High Avoidance X Loneliness, High Avoidance, Loneliness	.882 [.845–.921]	<.001*	−.125

Low Avoidance = 1SD below mean centered avoidance score, High Avoidance = 1SD above mean centered avoidance score.

Higher avoidant attachment was significantly associated with lower sleep quality (OR =.994, 95% CI [.989, 1.000], B = −.006, p = .033), as was higher anxious attachment (OR =.974, 95% CI [.970,.978], B = −.027, p < .001). Higher loneliness was associated with lower sleep quality (OR =.888, 95% CI [.873,.904], B = −.118, p < .001). Neither the avoidant attachment x loneliness interaction nor the anxious attachment x loneliness interaction was significant for sleep quality in the adjusted model (see Table 3).

**Table 3:** Predictor effects on sleep quality.

Predictor	OR [95% CI]	*p*	B
Age	1.000 [.998–1.003]	.795	.000
Gender (Men)	1.094 [1.008–1.187]	.031*	.090
women	1 ref.	ref.	ref.
Participation End Date	1.003 [1.001–1.005]	<.001*	.003
Avoidant Attachment	.994 [.989–1.000]	.033*	−.006
Anxious Attachment	.974 [.970–.978]	<.001*	−.027
Loneliness	.888 [.873–.904]	<.001*	−.118
Loneliness x Avoidant Attachment	.999 [.997–1.001]	.292	−.001
Loneliness x Anxious Attachment	1.001 [.999–1.002]	.520	.001

Higher avoidant attachment was significantly associated with longer sleep onset (OR = 1.006, 95% CI [1.001, 1.012], B = .006, p = .018), as was higher anxious attachment (OR = 1.020, 95% CI [1.016, 1.025], B = .020, p < .001). Higher loneliness was associated with longer sleep onset (OR = 1.066, 95% CI [1.049, 1.084], B = .064, p < .001). Neither the avoidant attachment x loneliness interaction nor the anxious attachment x loneliness interaction was significant for sleep onset in the adjusted model (see Table 4).

**Table 4:** Predictor effects on sleep onset.

Predictor	OR [95% CI]	*p*	B
Age	.996 [.994–.999]	.002*	−.004
Gender (Men)	.655 [.603–.711]	<.001*	−.424
women	1 ref.	ref.	ref.
Participation End Date	.997 [.995–.998]	<.001*	−.003
Avoidant Attachment	1.006 [1.001–1.012]	.018*	.006
Anxious Attachment	1.020 [1.016–1.025]	<.001*	.020
Loneliness	1.066 [1.049–1.084]	<.001*	.064
Loneliness x Avoidant Attachment	.999 [.998–1.001]	.592	−.001
Loneliness x Anxious Attachment	1.001 [.999–1.003]	.276	.001

Finally, higher anxious attachment and loneliness were significantly associated with longer wake-after-sleep onset (anxious attachment: OR = 1.014, 95% CI [1.010, 1.019], B = .014, p < .001; loneliness: OR = 1.062, 95% CI [1.045, 1.079], B = .060, p < .001). Avoidant attachment was not significantly associated with wake-after-sleep onset in the adjusted model (OR = 1.003, 95% CI [.998, 1.008], B = .003, p = .204). Neither the avoidant attachment x loneliness interaction nor the anxious attachment x loneliness interaction was significant for wake-after-sleep onset in the adjusted model (see Table 5).

**Table 5:** Predictor effects on wake-after-sleep onset.

Predictor	OR [95% CI]	*p*	B
Age	1.024 [1.022–1.027]	<.001*	.024
Gender (Men)	.687 [.636–.743]	<.001*	−.375
women	1 ref.	ref.	ref.
Participation End Date	.999 [.997–1.001]	.283	−.001
Avoidant Attachment	1.003 [.998–1.008]	.204	.003
Anxious Attachment	1.014 [1.010–1.019]	<.001*	.014
Loneliness	1.062 [1.045–1.079]	<.001*	.060
Loneliness x Avoidant Attachment	1.000 [.998–1.002]	.847	.000
Loneliness x Anxious Attachment	.999 [.998–1.001]	.537	−.001

## 4. Discussion

Previous studies have delineated the discrete impacts of attachment style and loneliness on various dimensions of sleep variables, wherein heightened levels of loneliness and the manifestation of both avoidant and anxious attachment styles have been associated with poorer sleep outcomes [9,12,38]. Still, the interrelationship between attachment style, loneliness and sleep remains unclear. In pursuit of a deeper understanding, the present investigation aimed to explore the interplay between attachment dimensions and loneliness, specifically how insecure attachment dimensions might moderate the relationship between loneliness and sleep patterns (sleep duration, perceived sleep quality, latency to sleep onset, and the temporal duration participants remained awake after nocturnal awakenings). The current study provides limited evidence that avoidant attachment may moderate the relationship between loneliness and one sleep outcome: sleep duration. Furthermore, while most studies of attachment and sleep focus on attachment styles [12], the current study used a dimensional approach to measuring attachment orientation, an approach proposed by adult attachment experts to be better suited to measuring individual differences than the categorical approach (Fraley et al. [19]).

Prior research suggests a relationship between secure attachment and diminished levels of loneliness [13]. Consequently, the current study hypothesised a positive correlation between heightened scores on anxious and avoidant attachment dimensions and elevated levels of loneliness. The findings of this investigation support the hypothesis, showing a statistically significant positive correlation between anxious attachment and loneliness and between avoidant attachment and loneliness. A proportion of the variance in loneliness – up to 17.6% – was ascribed to the avoidant attachment dimension in the current study, while up to 13% was related to the anxious attachment dimension.

It is important to acknowledge the findings of existing literature regarding the relationship between anxious and avoidant attachment styles and loneliness, as incongruent findings have been documented. For instance, studies such as that conducted by Ilhan [26] have reported mixed results, suggesting a significant correlation of loneliness exclusively with anxious attachment (not avoidant attachment). Another study found a correlation between both anxious and avoidant attachment and loneliness. However, the correlation and mean score of romantic loneliness were higher for those high in avoidant attachment [41]. However, Bernardon et al. [41] also found that individuals high in avoidant attachment had higher scores of family loneliness, suggesting that the association between attachment styles and loneliness may differ based on types of loneliness. Our study, with a substantially larger sample size, extends this literature by showing that both avoidant and anxious attachment dimensions appear to be associated with higher scores of loneliness.

The current study, however, does not distinguish between possible types of loneliness, such as emotional and social loneliness [42]. This heterogeneity in findings underscores the complexity of the interplay between attachment dimensions and the experience of loneliness. One factor that studies agree on however, is that insecure attachment in general (avoidant and anxious) appears to correlate with increased loneliness. Moving forward, while acknowledging differences between anxious and avoidant attachment in terms of loneliness, it may be worthwhile to consider insecure attachment as a whole compared to secure attachment.

Due to previously reported links between attachment styles and loneliness [13], and the links between both insecure attachment styles and loneliness with sleep [9,12], it was hypothesised that higher levels of each insecure attachment dimension (anxious and avoidant) would significantly moderate the relationship between loneliness and sleep (sleep quality, sleep duration, sleep onset, and wake-after-sleep onset). While controlling for age, gender, and participation end date, there was only a significant moderation effect of the avoidant attachment dimension on the relationship between loneliness and sleep duration. Increased loneliness was associated with decreased sleep duration at higher levels of the avoidant attachment dimension but not at lower levels. This interaction remained significant after false discovery rate correction and in a sensitivity analysis excluding participants who reported sleeping more than nine hours. However, the moderation hypothesis was not supported for sleep quality, sleep onset, or wake-after-sleep onset, suggesting that the interaction was specific to sleep duration rather than consistent across sleep outcomes. Our findings, in terms of avoidant attachment, are consistent with previous theories that suggest individuals with higher avoidant attachment might have heightened responses to loneliness due to certain mechanisms, such as maladaptive schema [27,43]. However, further research would need to be conducted to see if the mechanisms mentioned in previous literature play a role in the relationship between attachment, loneliness, and sleep.

These findings align with the work of Pietromonaco and Collins [8] by showing how feelings of social disconnection may be negatively affecting health outcomes, in this case sleep, while additionally suggesting that an avoidant attachment style might be acting as a potential moderator of the relationship. Later work by Pietromonaco and Beck [23] further suggests that this may be due to how attachment styles are related to affect regulation, managing our behaviour, and managing stress responses. It may be that individuals with higher levels of avoidant attachment might experience increased negative affect and stress in relation to higher feelings of loneliness, which in turn negatively affects sleep. While avoidantly attached individuals have a tendency to avoid intimacy, they may not deactivate their attachment systems and needs to the point of accepting a lack of supportive relationships, thus still negatively experiencing loneliness [28].

It was also hypothesised that the anxious attachment dimension might moderate the relationship between loneliness and sleep variables, with loneliness being associated with lower sleep quality in general at higher levels of anxious attachment. However, our findings do not provide support for this hypothesis. Our findings may be explained by the theories of Mikulincer and Shaver [28], who suggest that while both insecure attachment styles tend to be associated with increased loneliness, individuals with an avoidant attachment style have a lower drive to overcome loneliness and remain lonely over time. A drive for overcoming loneliness and finding social support is of importance as there have been associations between lack of support, loneliness, negative affect, and chronic interpersonal stress [44]. It may be that increased motivation for social support might act as a buffer in the loneliness-sleep relationship for individuals high in anxious attachment.

The absence of significant moderation effects for sleep quality, sleep onset, and wake-after-sleep onset might suggest the inclusion of factors beyond those measured here for future research. One possibility is that loneliness and attachment-related insecurity may be more strongly reflected in overall sleep quantity than in specific subjective indicators of sleep continuity or initiation in this sample. Sleep duration may capture a broader behavioural pattern, including delayed bedtimes, early waking, or reduced opportunity for sleep, whereas sleep onset and wake-after-sleep onset reflect more specific aspects of the sleep process. It is also possible that perceived sleep quality, sleep onset, and wake-after-sleep onset are more strongly influenced by unmeasured factors such as mental health symptoms, daily stress, sleep disorders, work schedules, medication use, or the sleep environment [e.g., 3, 4]. Because the current study relied on secondary, self-reported data, these mechanisms could not be tested directly. Therefore, the lack of findings across these outcomes should not be taken as evidence that attachment and loneliness are unrelated to these dimensions of sleep, but rather that their interactive effect was not detectable in the present models.

Past studies have found associations between increased loneliness and lower sleep quality, yet many mechanisms of the relationship are still unknown [9]. The current study suggests that avoidant attachment might act as a potential moderator of the loneliness-sleep duration relationship, although this finding was small in magnitude and should be interpreted cautiously.

### 4.1. Limitations and further research

One of the advantages of the current study is the large sample size, which increased the robustness of the findings. Furthermore, the study relied on reliable measures of attachment dimensions and loneliness. Nevertheless, the current study had some limitations. Firstly, participants were self-selected, which may have led to a non-representative UK sample and a possible risk of bias due to the possible interests of the participants. The current research is generalizable to a healthy UK sample and it is possible that the results may have varied for other countries. A healthy population was selected as certain mental health problems, such as depression, may have influenced the results. Due to the research’s cross-sectional and correlational nature, it is not possible to determine a causal relationship between the variables or the directions of the relationships. Using secondary data means that certain questions could not be asked of participants. Important potential confounding variables were unavailable or could not be modelled, including depression and anxiety symptoms, relationship status, socioeconomic variables, cohabitation, co-sleeping arrangements, and diagnosed sleep disorders. The sleeping environment of the participant is unknown, and certain factors, such as cohabiting and sleeping next to a partner, may influence sleep outcomes. While the current study measures relationship attachment, it would have been useful to know whether participants were currently in a relationship to see if the relationship status or sleeping next to a partner influenced the results. Unfortunately, the original data set did not include a question to ask participants about their relationship status. Complete-case analysis was used, and the analytic sample varied slightly by outcome because of missing sleep data. Complete-case analysis may introduce bias if data are not missing completely at random, and we therefore note that future work should consider multiple imputation or other missing-data approaches where appropriate.

Future work should examine whether missingness in large secondary datasets is associated with participant characteristics and should consider multiple imputation where appropriate. Follow-up studies should examine whether the possible absence of a partner might increase the moderation effect of anxious attachment on the sleep-loneliness relationship, as social support might be acting as a buffer in the relationship. As attachment styles become more activated and salient during difficult situations and increased stress, it may be possible that relationship factors and situational factors, such as the onset of COVID-19, may have influenced the self-reported attachment scores and sleep scores [20]. Based on the current analysis, while the participation end date did not influence loneliness, there appeared to be small associations between participation end date and both attachment dimensions, in addition to sleep quality, sleep onset, and wake after sleep onset, suggesting that the time of participation may have influenced the self-reported attachment scores and sleep scores. However, data collection was conducted before and during the COVID-19 pandemic and thus without a pandemic-related variable it is not possible to conclude if the pandemic was a significant factor in this study and influenced results. Finally, sleep duration was modelled as an ordered categorical outcome, although very long sleep may not necessarily represent better sleep health. To address this, we conducted a sensitivity analysis excluding participants who reported sleeping more than nine hours, and the key avoidant attachment x loneliness interaction remained significant.

In the future, it would be beneficial to conduct an experimental study on how increases and decreases in loneliness and attachment variables influence sleep variables. It would also be interesting to examine how other relationship variables (e.g., relationship satisfaction), emotional intelligence, or relationship demographics (e.g., relationship status), that have been suggested to be important in these relationships [43], may be related to the interaction effects of attachment and loneliness on sleep. It may also be that the moderation of insecure attachment of the relationship between loneliness and sleep may be heightened in times of relationship conflict or distress, which is a study currently under development by the researchers of this study. Dyadic studies that focus on both partners and their attachment style, loneliness, sleep, and possibly other personality factors such as resilience could also be fruitful and important directions for future research.

To conclude, avoidant attachment orientation may moderate the relationship between loneliness and sleep duration. Individuals high on the avoidant attachment dimension appeared more likely to report shorter sleep duration when they also reported higher loneliness. However, this moderation effect was small and was not observed for sleep quality, sleep onset, or wake-after-sleep onset. This pattern suggests that the combined effect of loneliness and avoidant attachment may be more relevant to sleep duration than to subjective sleep quality or specific markers of sleep initiation and maintenance, although further research using longitudinal and objective sleep measures is needed before firm conclusions can be drawn. Further investigations and interventions focused on improving individuals’ sleep should therefore consider relationship factors, such as attachment style and loneliness, while recognising that the present findings provide limited evidence for moderation across sleep outcomes. The current research provides an example of how attachment and relationship theory might relate to health outcomes with a focus on sleep; a health outcome of great importance to mental and physical health.
