# Supplementary material for: Adult attachment, loneliness, and self-reported sleep outcomes: A secondary analysis of the Touch Test Study
Source: PLoS One. 2026 Sep 8;21(9):e0356812. doi: 10.1371/journal.pone.0356812 (PMC13552711; doi:10.1371/journal.pone.0356812)
Supplement: Supplementary file 1 [file pone.0356812.s001.docx]

**Major comment 1. The manuscript’s main result is inconsistently reported across sections**

This is the most serious problem.

- On the **title-page abstract** (page 1), the submission text states that there were “increased odds of sleep quality in relation to the interaction of the anxious attachment domain and loneliness,” and also refers to significant interaction findings in the unadjusted model.
- In the **main abstract** (page 8, Results paragraph), only one interaction result is reported: higher loneliness associated with shorter sleep duration at higher levels of avoidant attachment.
- In the **main results** (pages 18–20), only the loneliness × avoidant attachment interaction for **sleep duration** is significant; the interaction terms for sleep quality, sleep onset, and WASO are not significant in the appendix tables.
- In the **final conclusion** (page 25), the manuscript states that avoidant attachment may moderate the relationship between loneliness and **sleep quality**, but the reported significant interaction is for **sleep duration**, not sleep quality.

This must be corrected throughout. As written, the paper does not present a stable central result.

**Required revision:**
Please align the title-page abstract, manuscript abstract, results, discussion, conclusion, tables, and figure legend so they all reflect exactly the same adjusted analyses. If unadjusted models were examined, report them clearly and separately rather than blending them into the narrative.

**Major comment 2. The modeling of sleep duration is not well justified**

The manuscript treats sleep duration as an ordered ordinal outcome from “less than 5 hours” up to “more than 9 hours” and interprets higher categories as better/longer sleep. That is a major substantive concern because sleep duration is not necessarily monotonic in health relevance; very long sleep can also be associated with poorer health outcomes. The current model implicitly assumes that moving from <5 to >9 hours lies on a single favorable continuum, which is not obviously defensible. The outcome definition is described on page 16, and the modeling approach is described on pages 16–17.

Because the paper’s headline result is the only significant interaction and it is on sleep duration, this issue is not peripheral; it affects the core claim.

**Required revision:**
Please justify this ordinal specification explicitly, or preferably reanalyze sleep duration using a more clinically interpretable approach, such as:

- multinomial categories (short / recommended / long), or
- a binary short-sleep outcome if that is the substantive focus.

At minimum, a sensitivity analysis is needed to show that the reported interaction is robust to a different coding of sleep duration.

**Major comment 3. Multiple testing is not addressed, and the key interaction is statistically fragile**

The study tests two interaction terms across four sleep outcomes, in addition to multiple main effects and several preliminary analyses. Yet the manuscript does not discuss family-wise error or false discovery control. The only significant moderation effect in the adjusted models appears to be loneliness × avoidant attachment on sleep duration with **p = .03** and **OR = .998**, which is extremely small in magnitude.

Given the number of tests performed, it is not clear whether this finding would remain significant after any reasonable multiplicity adjustment. Even if it does, the effect size needs much more careful interpretation.

**Required revision:**
Please:

1. state how many primary interaction tests were prespecified,
2. clarify whether this was a confirmatory or exploratory interaction analysis,
3. apply a multiplicity correction or justify why one is not needed,
4. interpret the practical significance of OR = .998 carefully rather than only the p-value.

**Major comment 4. The methods and results do not fully match**

On page 16, the methods state that the models included “the interaction of both attachment dimensions” as an independent variable, in addition to the two loneliness interaction terms. However, the tables do not display an anxious × avoidant attachment term.

This discrepancy matters because it changes the model being estimated.

**Required revision:**
Please clarify the exact model formula for each outcome. A transparent specification would help, for example:

Outcome ~ avoidant + anxious + loneliness + avoidant*loneliness + anxious*loneliness + age + gender + participation_end_date

If an anxious × avoidant term was included, it must be reported in the tables. If it was not included, the methods should be corrected.

**Major comment 5. Missing data handling is underreported and may bias the findings**

The manuscript begins with **15049** participants in Methods, while the abstract reports **15045**. The correlations are based on sample sizes around **11989–12087**, and the ordinal models use **11973** or **11910** participants. The manuscript states that only participants who completed all loneliness and attachment items were included and that missing data were excluded, but it does not provide a flow of exclusions or compare included vs excluded participants.

With complete-case analysis on a large secondary survey, this reporting is not sufficient.

**Required revision:**
Please provide:

- a participant flow table,
- the number excluded at each stage,
- reasons for exclusion where possible,
- a comparison of complete-case vs excluded participants on key demographics,
- justification for complete-case analysis rather than multiple imputation or another missing-data approach.

**Major comment 6. The choice of covariates appears data-driven rather than theory-driven**

The manuscript says that age, gender, and participation end date were included as covariates because preliminary analyses found associations with some variables. That is a data-driven selection strategy. Covariates in observational models should generally be chosen based on substantive theory, prior literature, or a causal diagram rather than only bivariate significance screens. The relevant description appears on pages 16–18.

In addition, important confounders are either unavailable or unmodeled, especially:

- depression/anxiety symptoms,
- relationship status,
- co-sleeping or cohabitation,
- socioeconomic variables.

The authors acknowledge some of these limitations later, but the discussion currently does not go far enough in recognizing the potential for residual confounding.

**Required revision:**
Please justify covariate selection more rigorously, and clarify which potentially important confounders were unavailable in the secondary dataset.

**Major comment 7. The paper says the predictions were preregistered, but the preregistration is not adequately reported**

The introduction states that the paper tested “pre-registered predictions” on page 13. However, the methods do not provide a preregistration DOI/URL for the analysis plan, registration date, or a statement of deviations from the preregistered plan. The OSF link provided on page 14 appears to be for questionnaire materials, not clearly the preregistration of the current secondary analysis.

**Required revision:**
Please provide:

- the exact preregistration link for this analysis,
- the date of preregistration,
- which hypotheses and analyses were preregistered,
- any deviations from the original plan.

If the study was not actually preregistered in the formal sense, the manuscript should not use that language.

**Major comment 8. Measures need clearer psychometric and scoring justification in the present sample**

The measures section is generally readable, but several details are missing or unclear.

1. **Attachment scoring**:
   The phrase “summed scores where means were calculated and then multiplied by the number of items” is confusing. Please explain the scoring algorithm clearly and state the possible score range.
2. **Loneliness measure**:
   The manuscript cites Russell et al. (1980), which is the classic UCLA loneliness paper, but the study uses a 4-item version. The exact short-form source and validation should be specified. Also, please report internal consistency in the current analytic sample rather than only published alpha values.
3. **Sleep variables**:
   These were “adapted” from the PSQI rather than taken as the full validated instrument. That weakens the claim of validated sleep measurement. Please avoid implying that full PSQI validity automatically transfers to selected/adapted items. Current-sample performance and coding direction should be reported explicitly.

**Major comment 9. Ordinal logistic regression assumptions and diagnostics are not reported**

The methods say that generalized linear models of an ordinal logistic type were used, but the manuscript does not report:

- whether the proportional odds / parallel lines assumption was tested,
- model fit statistics,
- threshold estimates/cutpoints,
- whether predictors were standardized,
- whether multicollinearity was assessed.

These are important for evaluating rigor, especially when conclusions rest on a very small interaction effect.

**Required revision:**
Please report the diagnostic checks for each ordinal model, or justify an alternative model if the assumptions were violated.

**Major comment 10. Data availability and ethics statements need harmonization**

There is a mismatch between the submission metadata and the manuscript text.

- In the submission pages, the authors indicate that the data are “fully available without restriction” and point to the UK Data Service link.
- In the manuscript conclusion section, the data availability statement says the dataset can be accessed “with permission from the original author” at the same link.

These are not equivalent statements, and PLOS ONE requires clarity here.

Similarly, the ethics statement in the submission system names the secondary-analysis approval code from the University of Bristol, but the main manuscript methods only state that ethical approval was provided by the University of Bristol without giving the approval number or clearly naming the original study approval body.

**Required revision:**
Please make the ethics and data availability statements fully consistent across the metadata and manuscript.

**Section-by-section comments**

**Title**

The title promises “effects on sleep quality,” but the paper actually examines four sleep outcomes, and the only significant interaction in the adjusted models concerns sleep duration rather than sleep quality. The title is therefore too narrow and somewhat misleading.

**Suggestion:** Consider a more neutral title such as:
“Adult attachment, loneliness, and self-reported sleep outcomes: A secondary analysis of the Touch-Test Study”

**Abstract**

**Page 8, all paragraphs; page 1 metadata abstract**

The abstract needs substantial revision.

- The sample size is inconsistent: 15045 in the abstract metadata vs 15049 in Methods.
- The main abstract reports only one significant moderation, but the title-page abstract reports additional interaction findings not supported by the appendix tables.
- The abstract does not mention the complete-case analytic sample size used in the models.
- The conclusion is too broad relative to the results.
- PLOS ONE abstracts should reflect the adjusted primary findings precisely and should not overgeneralize.

**Suggestion:** Rewrite the abstract around:

1. data source,
2. final analytic N for the main models,
3. exact significant and non-significant interaction findings,
4. a cautious conclusion.

**Introduction**

**Pages 9–13**

The introduction is generally coherent and gives reasonable background on attachment, loneliness, and sleep. The literature review is broad enough for a general audience. That said, several points need tightening:

1. The manuscript repeatedly suggests a mechanism whereby insecure attachment may affect sleep **through increased loneliness**, which is conceptually closer to **mediation**, yet the tested model is **moderation**. This theoretical shift is not explained clearly.
2. The REM-sleep biological discussion is interesting but quite speculative relative to what is actually tested here. Since the study is based on self-report survey items, I would recommend trimming or softening this mechanistic section.
3. The introduction says the study is testing preregistered predictions, but the preregistration itself is not properly introduced or linked as noted above.
4. Hypothesis 2 is very broad: it predicts moderation across all four outcomes and both attachment dimensions. Given that only one interaction appears significant, the discussion should explicitly frame this as largely unsupported overall.

**Suggestion:** Distinguish clearly between moderation and mediation at the theory stage.

**Methods**

**2.1 Data collection**

**Page 14, first two paragraphs**

This section is understandable, but it would help to specify:

- whether this was a new preregistered secondary analysis,
- what date the secondary analysis plan was finalized relative to data access,
- whether the current authors had access to the full original dataset or a restricted subset.

**2.2 Study participants**

**Page 14, “Study Participants” paragraph**

This section needs more justification.

- Excluding non-UK participants because other countries had lower representation is understandable statistically, but it should be framed as an analytic restriction rather than a theoretically motivated population definition.
- Excluding participants who selected gender as “other” is especially sensitive and should be justified carefully. At minimum, the manuscript should explain whether exclusion was due to extremely small cell sizes and inability to estimate stable effects, rather than treating the category as analytically irrelevant.
- The term “no psychological or physical health condition” needs an operational definition. Was this self-reported? Which items were used? Were sleep disorders included among exclusions?

**2.2.1 Participant characteristics**

**Pages 14–15**

Please report the characteristics of the final analytic sample for each model more transparently, not just the starting sample. A supplemental table is fine, but the main text should make clear that the effective N is closer to ~11,900 for the main regressions.

**2.3 Measures**

**Pages 15–16**

See Major comment 8. I would also like to see:

- the exact item wording for the adapted sleep items in supplement,
- score ranges for all predictors,
- current-sample alpha/omega where applicable,
- coding direction of each outcome category.

**2.4 Analysis**

**Page 16**

This section is currently too brief for the complexity of the models.

Please specify:

- exact model family and link,
- category ordering for each outcome,
- whether proportional odds assumptions were tested,
- whether interactions were prespecified,
- whether all continuous variables were centered only or also standardized,
- whether the reported ORs correspond to one-unit increases in raw scores,
- whether the simple slopes were estimated from the same ordinal models.

**2.5 Power analysis**

**Page 17**

I did not find this section persuasive in its current form.

- The study already has a large secondary sample, so this reads more like a retrospective justification than a design-based sample size calculation.
- The use of **r = .45** drawn from literature on attachment and mental health does not seem closely tied to the present interaction models and sleep outcomes.
- If the key interaction is very small, the power section should not substitute for careful interpretation of effect size.

I suggest shortening this section and focusing instead on transparent reporting of the realized sample and precision of estimates.

**Results**

**3.1 Preliminary analysis**

**Pages 17–18**

This section should be tightened.

- There appears to be a wording/sign inconsistency: the text says “higher age was associated with higher scores of anxious attachment” while reporting **r = -.13**. The direction described in words does not match the coefficient sign.
- Please avoid language such as “variance accounted for” when discussing squared bivariate rank correlations unless you want to be very precise about what is being summarized; this wording can imply a stronger explanatory interpretation than warranted in this context.

**3.2 Main analysis**

**Pages 18–20**

This is where the paper needs the most work.

1. The headline interaction is very small and should be interpreted cautiously.
2. The non-significant interactions should be reported more explicitly rather than submerged.
3. The results do not mention whether the models were significant overall or how much outcome variation they explained.
4. The simple slopes text appears to reverse the meaning of ±1 SD in one place. The manuscript says the effect was significant at higher avoidant attachment “(centered avoidant attachment - 1 SD)” and not at lower avoidant attachment “(+ 1 SD),” but Table 5 defines low avoidance as 1 SD below the mean and high avoidance as 1 SD above the mean. That is internally inconsistent.
5. Table 4 includes an apparent reporting error for the loneliness × anxious attachment term: **OR = .666** with **95% CI [1.000–.998]** and **p = .666**. That CI is impossible as written.

**Required revision:**
Please re-check all tables and narrative against the actual model output.

**Discussion**

**Pages 20–24**

The discussion has a reasonable structure, but it currently overstates the findings.

1. The paper broadly claims that loneliness and attachment insecurity negatively affect sleep outcomes, but the adjusted moderation evidence is limited to one small interaction for sleep duration.
2. The discussion sometimes slides from association language into quasi-mechanistic language that is not supported by the current cross-sectional self-report design.
3. The null findings for anxious attachment moderation deserve more emphasis.
4. The paper should explicitly say that the main preregistered moderation hypothesis was largely unsupported if that is the case after re-checking the models.

I recommend a more restrained discussion centered on:

- robust main effects vs fragile interaction evidence,
- limits of secondary cross-sectional inference,
- need for replication with better sleep measures and relationship-context variables.

**Limitations and conclusion**

**Pages 23–25**

The limitations section is one of the stronger parts of the manuscript because it acknowledges self-selection, cross-sectional design, lack of relationship-status data, and possible COVID timing effects.

However, the conclusion again repeats the wrong outcome: it says avoidant attachment may moderate loneliness in relation to **sleep quality**, whereas the significant adjusted interaction was for **sleep duration**.

This must be corrected.

**Tables and figure**

**Pages 30–34**

The appendix tables are useful, but they need technical cleanup.

- Table 4 contains a likely error in the CI for loneliness × anxious attachment.
- Table 5 and the text disagree on the sign/direction of high vs low avoidance.
- Figure 1 is readable, but its interpretation depends on the correct definition of high vs low avoidant attachment; please ensure the legend, text, and table all match.

I would also recommend adding a table of category coding for each sleep outcome.

**References and citation accuracy**

There are several reference-list problems that need correction.

Examples:

- **Glosemeyer et al. (2020)** is cited in the introduction but does not appear in the reference list.
- **Krizan et al.** appears as 2023 in text and 2024 in the reference list.
- **Xie & Feeney** appears as 2024 in text but 2022 in the references.
- **Pietromonaco & Beck** appears as 2018 in text but 2019 in the references.
- **Aanes et al.** appears as 2012 in text but 2009 in the references.

This needs a full audit before the manuscript can be evaluated reliably.

**Minor comments**

1. Please standardize the spelling of **Russell**; the manuscript uses “Russel” in the measures section.
2. Page 10: “Below we outline the attachment dimensions before also explaining how these map onto **an** category model...” should read “a category model.”
3. There are several formatting inconsistencies in p-values, spacing, and CI notation across the appendix tables.
4. Please ensure that all model terms are named consistently: “wake after sleep onset,” “wake-after-sleep onset,” and “wake after sleep” are all used.
5. The manuscript should specify whether “men” is the reference group or whether “women = 1 ref. ref.” is simply a table formatting artifact.
