# Supplementary material for: Adult attachment, loneliness, and self-reported sleep outcomes: A secondary analysis of the Touch Test Study
Source: PLoS One. 2026 Sep 8;21(9):e0356812. doi: 10.1371/journal.pone.0356812 (PMC13552711; doi:10.1371/journal.pone.0356812)
Supplement: Supplementary file 2 [file pone.0356812.s003.docx]

Response to Reviewer and Editor Comments

**Manuscript title:** Adult attachment, loneliness, and self-reported sleep outcomes: A secondary analysis of the Touch Test Study

**Manuscript ID:** PONE-D-26-05119

We thank the reviewer and editor for their careful and constructive feedback. We have revised the manuscript substantially and have provided a marked-up version showing changes from the previous submission. The main revisions include: aligning the central claim throughout the manuscript; revising the abstract, title, Results, Discussion, Conclusion, tables, and figure legend; clarifying the ordinal regression models and diagnostics; adding false discovery rate correction across the primary interaction tests; adding a sleep-duration sensitivity analysis; improving missing-data reporting; clarifying psychometric scoring and current-sample reliability; adding preregistration, ethics, and data availability details; and auditing the reference list and citation style.

# Responses to Major Comments

## Major comment 1. Main result inconsistently reported across sections

***Reviewer comment:*** *The reviewer noted that the title-page abstract, manuscript abstract, Results, Discussion, Conclusion, tables, and figure legend did not present a stable central result. In particular, the previous version sometimes referred to sleep quality, whereas the significant adjusted interaction concerned sleep duration.*

**Response:** We agree and have corrected this throughout the manuscript. The title, abstract, Results, Discussion, Conclusion, appendix tables, and figure legend now consistently state that the only significant attachment x loneliness interaction in the adjusted models was the avoidant attachment x loneliness interaction predicting sleep duration. The revised manuscript also states explicitly that the corresponding interactions were not significant for sleep quality, sleep onset, or wake after sleep onset. We removed language that blended adjusted and unadjusted findings and interpret the adjusted models as the primary analyses.

## Major comment 2. Modelling of sleep duration

***Reviewer comment:*** *The reviewer noted that treating sleep duration as an ordered ordinal outcome may be problematic because very long sleep is not necessarily more favourable than shorter sleep, and requested justification or sensitivity analysis.*

**Response:** We agree that sleep duration is not a straightforward monotonic health outcome. We retained the ordinal logistic specification because the original response options were ordered sleep-duration categories and to maintain comparability with prior literature using the PSQI sleep-duration component. However, we acknowledge that very long sleep may not reflect a more favorable sleep outcome. To address this concern, we conducted a sensitivity analysis excluding participants reporting more than nine hours of sleep per night. This group comprised only 0.7% of the sample (n = 113). The avoidant attachment × loneliness interaction showed the same pattern and remained significant in this sensitivity analysis (OR = .997, 95% CI [.995, .999], B = -.003, p = .002). We have revised the Results and Limitations sections accordingly and now interpret the sleep-duration findings more cautiously.

**Major comment 3. Multiple testing and practical significance of the key interaction**

***Reviewer comment:*** *The reviewer noted that two interaction terms were tested across four sleep outcomes and requested clarification of the number of primary interaction tests, multiplicity correction, and cautious interpretation of the small interaction effect.*

**Response:** We have revised the Analysis and Results sections to state that the primary focus was on two attachment x loneliness interaction terms across four sleep outcomes, resulting in eight primary interaction tests. We applied Benjamini-Hochberg false discovery rate correction across these eight interaction tests. The avoidant attachment x loneliness interaction predicting sleep duration remained significant after correction (FDR-adjusted p = .020). We also revised the Abstract, Results, and Discussion to describe the effect as limited and small in magnitude rather than emphasising statistical significance alone. We interpret OR = .998 as a per-unit raw-score effect" so we might want to say that in the letter as it also speaks to practical signifance. For instanace, "We also note that the reported odds ratios correspond to one-unit increases in the raw-score interaction term. Because the interaction term is derived from the product of two continuous variables, a one-unit increase represents a relatively small change on the underlying scale. Accordingly, odds ratios close to 1.00 should not be interpreted as necessarily indicating an absence of association. We have not added a detailed discussion of this point to the manuscript, but note it here in response to the reviewer’s query. In the revised manuscript, we instead focus on the limited magnitude of the observed association and avoid overemphasising statistical significance. We have also specified that our analyses were hypothesis-driven and specified a priori. Pre-registration links have been added to the manuscript.

## Major comment 4. Methods and Results mismatch

***Reviewer comment:*** *The reviewer noted that the previous Methods section referred to “the interaction of both attachment dimensions,” whereas the tables did not include an anxious attachment x avoidant attachment term.*

**Response:** We have corrected the Methods section to specify the exact model estimated for each outcome. The revised model includes avoidant attachment, anxious attachment, loneliness, the avoidant attachment x loneliness interaction, the anxious attachment x loneliness interaction, age, gender, and participation end date. We also added the model formula in the Analysis section for transparency.

## Major comment 5. Missing data handling and complete-case analysis

***Reviewer comment:*** *The reviewer requested clearer reporting of missing data, including a participant flow table, number excluded at each stage, reasons for exclusion, comparison of complete-case and excluded participants, and justification of complete-case analysis.*

**Response:** We have expanded the missing-data reporting. The revised manuscript now reports the final complete-case sample sizes for each model: N = 11,914 for sleep duration, N = 11,916 for sleep quality, N = 11,908 for sleep onset, and N = 11,901 for wake after sleep onset. The Supplementary Material now includes a participant exclusion flow table showing the full Touch Test dataset, sex/gender restriction, Healthy UK analytic restriction, and complete-case samples for each sleep model. We also added a comparison of participants included versus excluded from the sleep-duration model on age, gender, and ethnicity.

Complete-case analysis is now described explicitly in the Analysis and Limitations sections. We retained a complete-case approach because this was a secondary analysis of an existing dataset, the resulting analytic samples remained large, and missingness was primarily item-specific. We acknowledge, however, that complete-case analysis may introduce bias if data are not missing completely at random, and we therefore note that future work should consider multiple imputation or other missing-data approaches where appropriate.

## Major comment 6. Covariate selection and residual confounding

***Reviewer comment:*** *The reviewer noted that covariate selection appeared data-driven and asked for stronger theoretical justification and clearer acknowledgement of unavailable confounders, including depression/anxiety symptoms, relationship status, co-sleeping/cohabitation, and socioeconomic variables.*

**Response:** We have revised the Analysis section to justify covariate selection more clearly and no longer describe covariate inclusion as being based solely on preliminary statistical associations. Age and gender are now described as retained because both are relevant to sleep and psychosocial outcomes in prior research, and participation end date was retained to account for timing effects in the Touch Test dataset and the early COVID-19 period. We also expanded the Limitations section to state that important potential confounders were unavailable or could not be modelled, including depression and anxiety symptoms, relationship status, socioeconomic variables, cohabitation, co-sleeping arrangements, and diagnosed sleep disorders. We now explicitly acknowledge the possibility of residual confounding.

## Major comment 7. Preregistration reporting

***Reviewer comment:*** *The reviewer requested the exact preregistration link, date of preregistration, statement of what was preregistered, and deviations from the original plan.*

**Response:** We have revised the Data Collection section to include the preregistration date and archived link. The manuscript now states that the study was preregistered on October 10th 2023 prior to data analysis and gives the archived preregistration link. The preregistration specified analyses of all interactions and covariates in addition to hypotheses that suggest direction and significance. We also added a statement of deviation from the preregistered analysis plan: the three-way interaction between attachment anxiety, attachment avoidance, and loneliness was omitted because the revised analysis focused on the two theoretically specified attachment x loneliness interactions.

## Major comment 8. Psychometric and scoring details for measures

***Reviewer comment:*** *The reviewer requested clearer attachment scoring, possible score ranges, current-sample reliability for loneliness, and clarification that the sleep variables were adapted PSQI items rather than the full PSQI.*

**Response:** We have revised the Measures section. For attachment, we now explain that attachment anxiety and attachment avoidance scores were calculated separately by averaging valid responses within each six-item subscale and multiplying the mean by six, producing scores from 6 to 42. We report current-sample internal consistency for attachment anxiety (alpha = .85) and attachment avoidance (alpha = .87). For loneliness, we now report that total scores ranged from 4 to 16 and that current-sample reliability was acceptable (alpha = .73). We have revised the Measures section to specify that loneliness was assessed using a 4-item version of the UCLA Loneliness Scale rather than the full 20-item measure. We now cite the psychometric evaluation supporting this. This was conducted using the Touch Test dataset (Panayiotou et al., 2022), which identified this 4-item version as a robust and reliable measure of loneliness across adult age groups. We also describe the items included in the scale, report the score range (4–16), and provide current-sample internal consistency (α = .73)."

For sleep, we clarify that the outcomes were selected items adapted from the PSQI and were analysed as separate self-reported sleep outcomes rather than as a composite PSQI score.

## Major comment 9. Ordinal logistic regression diagnostics

***Reviewer comment:*** *The reviewer requested diagnostic checks for the ordinal models, including the proportional odds/parallel lines assumption, model fit statistics, thresholds/cutpoints, centering/standardisation, and multicollinearity.*

**Response:** We have revised the Analysis and Results sections and added model diagnostics in the Supplementary Material. The revised Analysis section now states that predictors were mean-centred before interaction terms were created and that multicollinearity was assessed using variance inflation factors, all below 1.40. The Supplementary Material reports model fit statistics, Nagelkerke R-squared values, Pearson and deviance goodness-of-fit tests, and tests of the proportional odds assumption for all four ordinal logistic regression models. Because the test of parallel lines was significant for most sleep measures, the Results and Discussion now state that the findings should be interpreted cautiously. However, we retained the ordinal specification because it reflects the original ordered response categories and because sensitivity analyses yielded substantively similar conclusions.

## Major comment 10. Data availability and ethics statements

***Reviewer comment:*** *The reviewer noted that the data availability and ethics statements were inconsistent with the submission metadata and requested harmonisation.*

**Response:** We have revised both statements. The Data Availability Statement now states that the data underlying this study are available from the UK Data Service ReShare repository, identifies the dataset as The Touch Test: Cross-sectional survey data, 2020, and provides the direct access link. We removed wording implying that the dataset is available only with permission from the original author. The ethics statement now identifies the University of Bristol School of Psychological Science Research Ethics Committee, the approval date (03/11/2023), and ethics approval code 16127.

# Responses to Section-by-Section Comments

## Title

***Reviewer comment:*** *The reviewer suggested that the previous title was too narrow and misleading because it promised effects on sleep quality, whereas the paper examined four sleep outcomes and the key adjusted interaction concerned sleep duration.*

**Response:** We changed the title to a neutral title reflecting the full scope of the study: “Adult attachment, loneliness, and self-reported sleep outcomes: A secondary analysis of the Touch Test Study.”

## Abstract

***Reviewer comment:*** *The reviewer requested substantial revision of the abstract, including consistent sample size, final analytic N, exact significant and non-significant interaction findings, and a cautious conclusion.*

**Response:** We rewrote the abstract. It now reports the eligible sample (N = 15,049), the final analytic sample range across the main models (N = 11,901 to N = 11,916), the only significant adjusted interaction (avoidant attachment x loneliness predicting sleep duration), and the non-significant interactions for sleep quality, sleep onset, and wake after sleep onset. The conclusion now describes the findings as limited evidence and notes that the observed moderation effect was specific to sleep duration and small in magnitude. The online submission abstract will be updated to match the manuscript abstract exactly.

## Introduction

***Reviewer comment:*** *The reviewer recommended distinguishing moderation from mediation, trimming speculative REM-sleep biological discussion, clarifying preregistration, and framing the broad moderation hypothesis cautiously.*

**Response:** We revised the Introduction to focus on psychological and behavioural pathways linking attachment, loneliness, and sleep, rather than REM-sleep biological mechanisms that were not directly tested. The speculative REM paragraph was removed. We clarified the rationale for moderation by explaining that the association between loneliness and sleep may depend on attachment-related individual differences. We also removed unsupported preregistration phrasing from the Introduction and added preregistration details in the Methods. The Discussion now explicitly states that the moderation hypothesis was not supported across most sleep outcomes.

## Methods: Data collection

***Reviewer comment:*** *The reviewer requested clarity on the preregistered secondary analysis, date of analysis plan, and dataset access.*

**Response:** We revised the Data Collection section to state that the current study was a secondary analysis of the Touch Test dataset, that the data were accessed on 16/10/2023, and that the study was preregistered on October 10th 2023 before data analysis. We also identify the blocks from the original questionnaire used in the secondary analysis: loneliness, attachment style, demographics, and sleep.

## Methods: Study participants

***Reviewer comment:*** *The reviewer asked for clearer justification of the analytic restrictions, including UK participants, men/women only, and no psychological or physical health condition.*

**Response:** We revised the Study Participants section to frame the UK and gender restrictions as analytic restrictions to maximise sample size and stable model estimation. The manuscript now explains that non-UK participants and participants selecting gender as “other” were excluded because representation from these groups was substantially lower, resulting in unstable cell sizes. The manuscript also clarifies that the analytic sample was restricted to participants from the UK with no self-reported physical or psychological health condition.

## Methods: Participant characteristics

***Reviewer comment:*** *The reviewer requested transparent reporting of final analytic sample sizes rather than only the starting sample.*

**Response:** We now report the final analytic sample sizes for each regression model in both the Abstract and Analysis sections. The Supplementary Material also provides baseline characteristics, participant exclusion flow, and comparison of included versus excluded participants.

## Methods: Measures

***Reviewer comment:*** *The reviewer requested exact item wording for adapted sleep items, score ranges, current-sample reliability, and coding direction.*

**Response:** We revised the Measures section to include score ranges for attachment and loneliness and to report current-sample reliability. The sleep section now describes the item wording and response categories for sleep duration, sleep quality, sleep onset latency, and wake after sleep onset. We clarify that higher sleep-duration categories indicate longer sleep duration, sleep-quality categories indicate better self-reported sleep quality, and higher sleep-onset and wake-after-sleep-onset categories indicate longer times.

## Methods: Analysis

***Reviewer comment:*** *The reviewer requested the exact model family and link, category ordering, proportional odds tests, interaction specification, centering/standardisation, OR interpretation, and simple slopes clarification.*

**Response:** We expanded the Analysis section. It now specifies four ordinal logistic regression models, the same adjusted model for each sleep outcome, the exact predictors and interaction terms, the complete-case criteria, and the final N for each model. Continuous predictors involved in interactions were mean-centred before interaction terms were created. We report VIFs and model diagnostics, including proportional odds tests, in the Supplementary Material. The simple slopes text has been corrected so that low avoidance is 1 SD below the mean and high avoidance is 1 SD above the mean.

## Methods: Power analysis

***Reviewer comment:*** *The reviewer noted that the power analysis read as a retrospective justification and requested more careful framing.*

**Response:** We revised the Power Analysis section to state that, because the study used a large secondary dataset, the power analysis should be interpreted as a guide to detectable interaction effect size rather than as a prospective design-based sample size calculation.

## Results: Preliminary analysis

***Reviewer comment:*** *The reviewer noted a wording/sign inconsistency for age and anxious attachment and suggested avoiding overly strong “variance accounted for” wording.*

**Response:** We corrected the direction of the age/anxious attachment association so that the text now states that age was negatively correlated with anxious attachment. We also checked the preliminary results against the output. We can further simplify the “variance accounted for” wording if requested, but the revised manuscript now avoids the previous sign error.

## Results: Main analysis

***Reviewer comment:*** *The reviewer requested more cautious interpretation of the small headline interaction, explicit reporting of non-significant interactions, model fit information, correction of the simple slopes direction, and correction of the Table 4 reporting error.*

**Response:** We revised the Main Analysis section extensively. The key interaction is now described as small and interpreted cautiously. The manuscript explicitly states that the attachment x loneliness interactions were not significant for sleep quality, sleep onset, or wake after sleep onset. Model fit and Nagelkerke R-squared for the sleep-duration model are now reported in the main Results, and full model diagnostics for all four outcomes are provided in the Supplementary Material. The simple slopes text has been corrected, and the erroneous Table 4 confidence interval has been corrected based on the rerun model output.

## Discussion

***Reviewer comment:*** *The reviewer noted that the Discussion overstated the findings and sometimes used mechanistic language not supported by the cross-sectional self-report design.*

**Response:** We revised the Discussion to focus on limited evidence for one moderation effect: avoidant attachment x loneliness predicting sleep duration. We now state explicitly that the moderation hypothesis was not supported for sleep quality, sleep onset, or wake after sleep onset. We also softened mechanistic language and state that the proposed psychological and behavioural mechanisms require further testing. The Discussion now emphasises the limits of the cross-sectional secondary survey design and the need for replication using longitudinal, dyadic, and more detailed sleep measures.

## Limitations and conclusion

***Reviewer comment:*** *The reviewer noted that the conclusion repeated the wrong outcome and should be corrected to sleep duration.*

**Response:** We corrected the Conclusion. It now states that avoidant attachment orientation may moderate the relationship between loneliness and sleep duration, not sleep quality. It also states that the moderation effect was small and not observed for sleep quality, sleep onset, or wake after sleep onset.

## Tables and figure

***Reviewer comment:*** *The reviewer noted a likely error in Table 4, disagreement between Table 5 and the simple slopes text, and the need to ensure Figure 1 matched the correct definition of high and low avoidance.*

**Response:** We checked the tables and figure against the updated model output. Table 4 was corrected, Table 5 and the text now consistently define low avoidance as 1 SD below the mean and high avoidance as 1 SD above the mean, and the Figure 1 caption was revised to clarify that higher values on the sleep-duration axis indicate longer sleep duration and that higher loneliness was associated with shorter sleep duration particularly at higher levels of avoidant attachment.

## References and citation accuracy

***Reviewer comment:*** *The reviewer identified several reference-list problems, including missing or mismatched references for Glosemeyer, Krizan, Xie and Feeney, Pietromonaco and Beck, and Aanes.*

**Response:** We conducted a full reference audit. The speculative REM paragraph was removed, so the Glosemeyer citation was no longer needed. The year mismatches for Krizan et al., Xie et al., Pietromonaco and Beck, and Aanes et al. were corrected. We also standardised the spelling of Russell and updated the manuscript to PLOS ONE numbered citation style and Vancouver-style reference formatting.

## Minor comments

***Reviewer comment:*** *The reviewer requested correction of Russell spelling, “an category model,” formatting inconsistencies in p-values and confidence intervals, consistent naming of wake after sleep onset, and clarification of the gender reference group.*

**Response:** We corrected the spelling of Russell, changed “an category model” to “a categorical model,” corrected formatting inconsistencies in the tables where possible, used consistent terminology for wake after sleep onset, and clarified in the tables that women are the reference group for gender.

# Responses to Editor Comments

## Editor comment 1. PLOS ONE style requirements and file naming

***Reviewer comment:*** *The editor asked that the manuscript meet PLOS ONE style requirements, including file naming.*

**Response:** We have reformatted the in-text citations and reference list to match PLOS ONE numbered/Vancouver style more closely. Files will be uploaded with clear names, including “Manuscript_Revised”, “Revised Manuscript with Track Changes”, “Supplementary_Methods”, and “Response_to_Reviewers”.

## Editor comment 2. Data Availability Statement

***Reviewer comment:*** *The editor asked for the repository name and DOI/accession number or direct link to the dataset.*

**Response:** We revised the Data Availability Statement to include the repository name and direct link: “The data underlying this study are available from the UK Data Service ReShare repository: The Touch Test: Cross-sectional survey data, 2020. The dataset can be accessed at https://reshare.ukdataservice.ac.uk/854471.”

## Editor comment 3. Online abstract and manuscript abstract must be identical

***Reviewer comment:*** *The editor noted that the abstract in the online submission form and the manuscript must be identical.*

**Response:** We will copy the revised manuscript abstract exactly into the online submission form during resubmission to ensure the two versions are identical.

## Editor comment 4. Reviewer-recommended citations

***Reviewer comment:*** *The editor clarified that suggested citations should be reviewed for relevance and are not required unless specifically requested by the editor.*

**Response:** We reviewed the references and retained or added only those citations that directly support the revised text. We removed the speculative REM-related material rather than adding a missing citation that was no longer necessary after revision.

We again thank the reviewer and editor for their helpful comments. We believe these revisions have improved the clarity, transparency, and caution of the manuscript substantially.
