# Supplementary material for: Adult attachment, loneliness, and self-reported sleep outcomes: A secondary analysis of the Touch Test Study
Source: PLoS One. 2026 Sep 8;21(9):e0356812. doi: 10.1371/journal.pone.0356812 (PMC13552711; doi:10.1371/journal.pone.0356812)
Supplement: Peer review history

# Peer review: Decision Letter 0

**DOI:** 10.1371/journal.pone.0356812.r001

**Authors:** Runtang Meng

14 May 2026

Dear Dr. Samii,

Thank you for submitting your manuscript to PLOS ONE. After careful consideration, we feel that it has merit but does not fully meet PLOS ONE’s publication criteria as it currently stands. Therefore, we invite you to submit a revised version of the manuscript that addresses the points raised during the review process.

Please submit your revised manuscript by Jun 28 2026 11:59PM. If you will need more time than this to complete your revisions, please reply to this message or contact the journal office at plosone@plos.org. When you're ready to submit your revision, log on to https://www.editorialmanager.com/pone/ and select the 'Submissions Needing Revision' folder to locate your manuscript file.

- A letter that responds to each point raised by the academic editor and reviewer(s). You should upload this letter as a separate file labeled 'Response to Reviewers'.
- A marked-up copy of your manuscript that highlights changes made to the original version. You should upload this as a separate file labeled 'Revised Manuscript with Track Changes'.
- An unmarked version of your revised paper without tracked changes. You should upload this as a separate file labeled 'Manuscript'.

If you would like to make changes to your financial disclosure, please include your updated statement in your cover letter. Guidelines for resubmitting your figure files are available below the reviewer comments at the end of this letter.

If applicable, we recommend that you deposit your laboratory protocols in protocols.io to enhance the reproducibility of your results. Protocols.io assigns your protocol its own identifier (DOI) so that it can be cited independently in the future. For instructions see: https://journals.plos.org/plosone/s/submission-guidelines#loc-laboratory-protocols. Additionally, PLOS ONE offers an option for publishing peer-reviewed Lab Protocol articles, which describe protocols hosted on protocols.io. Read more information on sharing protocols at https://plos.org/protocols?utm_medium=editorial-email&utm_source=authorletters&utm_campaign=protocols.

As the corresponding author, your ORCID iD is verified in the submission system and will appear in the published article. PLOS supports the use of ORCID, and we encourage all coauthors to register for an ORCID iD and use it as well. Please encourage your coauthors to verify their ORCID iD within the submission system before final acceptance, as unverified ORCID iDs will not appear in the published article. *Only*  the individual author can complete the verification step; PLOS staff *cannot*  verify ORCID iDs on behalf of authors.

We look forward to receiving your revised manuscript.

Kind regards,

Runtang Meng, PhD

Academic Editor

PLOS One

Journal Requirements:

When submitting your revision, we need you to address these additional requirements.

1. Please ensure that your manuscript meets PLOS ONE's style requirements, including those for file naming. The PLOS ONE style templates can be found at

https://journals.plos.org/plosone/s/file?id=wjVg/PLOSOne_formatting_sample_main_body.pdf and

https://journals.plos.org/plosone/s/file?id=ba62/PLOSOne_formatting_sample_title_authors_affiliations.pdf

2. Please note that your Data Availability Statement is currently missing the repository name and the DOI/accession number of each dataset or a direct link to access each database. If your manuscript is accepted for publication, you will be asked to provide these details on a very short timeline. We therefore suggest that you provide this information now, though we will not hold up the peer review process if you are unable.

3. Please amend either the abstract on the online submission form (via Edit Submission) or the abstract in the manuscript so that they are identical.

4. If the reviewer comments include a recommendation to cite specific previously published works, please review and evaluate these publications to determine whether they are relevant and should be cited. There is no requirement to cite these works unless the editor has indicated otherwise.

[Note: HTML markup is below. Please do not edit.]

Reviewers' comments:

Reviewer's Responses to Questions

**Comments to the Author**

1. Is the manuscript technically sound, and do the data support the conclusions?

Reviewer #1: Partly

Reviewer #2: Partly

Reviewer #3: Partly

Reviewer #4: Partly

**********

2. Has the statistical analysis been performed appropriately and rigorously?

Reviewer #1: Yes

Reviewer #2: No

Reviewer #3: Yes

Reviewer #4: No

**********

3. Have the authors made all data underlying the findings in their manuscript fully available?

The PLOS Data policy

Reviewer #1: Yes

Reviewer #2: Yes

Reviewer #3: Yes

Reviewer #4: Yes

**********

4. Is the manuscript presented in an intelligible fashion and written in standard English?

Reviewer #1: Yes

Reviewer #2: Yes

Reviewer #3: Yes

Reviewer #4: Yes

**********

Reviewer #1: 1. The authors are suggested to highlight in Abstract the novelty of the study and the main finding, especially the moderating role of avoidant attachment.

2. In Introduction Section, it is suggested to streamline the introduction to attachment theory, focusing specifically on its regulatory mechanisms concerning sleep and loneliness.

3. In Results Section, it is suggested to emphasize the magnitude of effect sizes and avoid overinterpreting statistical significance alone.

4. In Discussion Section, it is suggested to provide theoretical explanations for nonsignificant results and discuss the impact of sample bias on the findings.

5. Please ensure all tables are correctly cited within the text and include appropriate titles and notes.

Reviewer #2: This manuscript presents a secondary analysis of a large dataset and aimed at examining the moderating role of attachment style on the association between loneliness and sleep quality. The topic is relevant and underexplored, and the large sample size provides strong statistical power supporting its potential for publication.

However, several methodological and reporting issues should be addressed to strengthen the rigor and interpretation of the findings.

1- The manuscript does not report reliability indices (e.g., Cronbach’s alpha) for the scales used in the present sample. Even when validated instruments are employed, it is standard practice to report internal consistency within the study population to ensure reliability of the used scales, as psychometric properties may vary across samples. This is crucial is interpreting the results obtained in the analysis. If reliability isn’t supported, the robustness of the results is affected and this should be clearly mentioned and cautiously interpreted.

Recommendation: Please report Cronbach’s alpha (or other appropriate reliability measures) for all scales included in the analysis.

2- All key variables (loneliness, attachment style, and sleep quality) were assessed using self-report instruments. While this is common in large epidemiological studies, it introduces potential biases related to self-reporting, including recall bias and social desirability bias. This is particularly all the more relevant for sleep-related variables, as accurate recall of parameters such as sleep latency or nighttime awakenings may be limited.

Recommendation: The limitations section would benefit from mentioning this point.

3- The authors appropriately acknowledge the limited number of variables available due to the secondary nature of the dataset. However, the absence of key potential confounders remains an important limitation. While this constraint cannot be resolved, its implications should be more explicitly integrated into the interpretation of the findings, particularly regarding the strength and generalizability of the observed moderation effect (even withing the healthy UK population and despite the big sample size)

Recommendation: Emphasize the role of residual confounding more clearly in the discussion highlighting the literature studying potential missing confounders.

Reviewer #3: I appreciate the opportunity to review the work "Adult Attachment, Loneliness, and the Effects on Sleep Quality" which reports a secondary analysis of data from the Touch Test study (BBC/Wellcome Collection, 2020) to examine how the dimensions of adult attachment (anxiety and avoidance) and loneliness are associated and interact to influence sleep outcomes (duration, quality, onset latency and time of wakefulness after nighttime awakening).

The abstract adequately describes the study. However, the abstract on the cover page and the abstract in the body of the manuscript report different results. The former mentions a significant effect of the interaction between anxious attachment and loneliness on sleep quality; the latter does not report such an effect. The abstract in the body of the manuscript omits the significant main effects of both attachment and loneliness on all four sleep outcomes, which are consistent and robust findings of the study.

The introduction provides a well-constructed conceptual framework that contextualizes the clinical and social relevance of sleep, loneliness, and adult attachment as interrelated factors. The interaction between these variables has not been directly examined in previous studies, making this research compelling and original. However, the controversy over whether avoidant attachment protects or amplifies the loneliness stress response —central to the moderation hypothesis— is not developed in sufficient depth.

The objective is clear, although the overall objective could be stated more precisely by explicitly mentioning the hypothesized moderating role of the attachment dimensions.

Materials and Methods

There is inconsistency in the reported N (15,045 in the abstract, 15,049 in the text, ~11,970 in the models). A participant flowchart explaining the exclusions is suggested. The exact procedure for centering variables and creating interaction terms is mentioned but not detailed. It is not reported whether the proportionality of odds assumption (parallel lines test) required by ordinal logistic regression was verified. The description of the ECR-12 scoring method is ambiguous (attachment scores were calculated as 'summed scores where means were calculated and then multiplied by the number of items in each scale'). The 4-item UCLA-LS version lacks the correct citation; this version, Russell et al. (1980), corresponds to the original 20-item scale. The sleep items adapted from the PSQI lack psychometric support in the sample. Instead, the items adapted by Dueren et al. were used. (2022), without reporting evidence of validity or reliability.

The a priori power analysis is a notable strength of the manuscript, especially the use of InteractionPowerR() to estimate the N needed to detect small interaction effects. However, the result of the proportionality of odds test should be reported to validate the ordinal model, and the pseudo-R² values should be discussed as a complement to the ORs.

The results are presented in an orderly fashion. However, the odds ratios (ORs) for all significant effects are extremely close to 1.0, indicating practically trivial effect sizes. This situation—common with very large sample sizes—requires an explicit discussion distinguishing between statistical significance and practical relevance. Table 4 contains a typographical error in the confidence interval (CI) for the OR for Loneliness × Anxious Attachment, which should be corrected. The simple slope analysis (Table 5) is appropriate and supports the interpretation of moderation. However, the authors should also report whether the difference between the slopes at high and low levels of avoidance is statistically significant (test of difference of slopes).

The inclusion of 'participation end date' as a covariate is methodologically justified given that data collection took place during the COVID-19 pandemic. However, the authors note that this variable correlates with both dimensions of attachment and with three of the four sleep outcomes, suggesting that the collection period (pre-pandemic vs. pandemic) is a significant confounder. The dichotomous variable pre/during COVID-19 would have been more interpretable than a continuous date, and its moderating effect on the results warrants further discussion than is currently offered.

In Table 4 (Wake-After-Sleep Onset predictors), the OR for Loneliness×Anxious Attachment reports '[.666-1.000-.998]' which is clearly a typographical or formatting error.

The authors conclude that the results do not support H2 for moderation of the anxiety dimension. However, the discussion does not delve deeply enough into why this moderation was not significant or under what conditions it might be expected. The explanation offered (that anxious individuals might have greater motivation to overcome loneliness as a buffer) is speculative and not directly cited.

The final conclusion states that 'avoidant attachment orientation may moderate the relationship between loneliness and sleep quality'. However, the only outcome in which significant moderation was found was sleep duration, not sleep quality (Table 2: OR=.999, p=.329).

The results of the simple slope analysis (Table 5) indicate that the relationship between loneliness and sleep duration is significant only at high levels of avoidant attachment, but the direction is not sufficiently clear in the text. It is stated that 'higher values of loneliness and avoidant attachment indicate shorter sleep duration' (Figure 1 legend), but in Table 1 the OR for Avoidant Attachment = 0.985 (B = -.015) also indicates shorter duration with higher avoidance, even as a main effect.

Aanes et al. (2009) appears correctly in the reference list but in the text it is cited as Aanes et al. (2012)

Reviewer #4: This manuscript addresses an interesting and potentially important question: whether attachment dimensions and loneliness jointly relate to sleep outcomes in a large secondary dataset. The topic is appropriate for PLOS ONE, and the sample size is a strength. However, in its current form, the paper has several serious problems of internal consistency, model specification, reporting accuracy, and interpretation that prevent a full assessment of methodological rigor. The most important issue is that the manuscript’s central claim is not reported consistently across the title-page abstract, main abstract, results, discussion, conclusion, and tables. In addition, the analytic approach to sleep duration and the handling of multiple tests require substantial revision. The paper’s strongest features are the large dataset, use of established constructs, and an attempt to examine interaction effects rather than only main effects. However, I do not think the manuscript is ready for publication without major reanalysis and substantial rewriting. The key issues are:

1. Internal inconsistency of the main finding

2. Questionable modeling of sleep duration as an ordinal monotonic outcome

3. No clear control for multiplicity despite many tests

4. Insufficient reporting of missing data, analytic sample derivation, and model diagnostics

5. Methods and results do not fully match

6. Discussion and conclusion overstate the evidence

I think the question is publishable in principle, but the current manuscript needs major revision before it can be judged methodologically sound. The authors need to correct internal contradictions, clarify the preregistration and model specification, justify or reanalyze the sleep duration outcome, address multiplicity, and rewrite the discussion and conclusion so they match the actual findings.

**********

what does this mean?). If published, this will include your full peer review and any attached files.

If you choose “no”, your identity will remain anonymous but your review may still be made public.

**Do you want your identity to be public for this peer review?**  For information about this choice, including consent withdrawal, please see our Privacy Policy

Reviewer #1: No

Reviewer #2: No

Reviewer #3: **Yes:**  Francisco Paz Rodríguez

Reviewer #4: **Yes:**  Mieska Despitasari

**********

[NOTE: If reviewer comments were submitted as an attachment file, they will be attached to this email and accessible via the submission site. Please log into your account, locate the manuscript record, and check for the action link "View Attachments". If this link does not appear, there are no attachment files.]

To ensure your figures meet our technical requirements, please review our figure guidelines: https://journals.plos.org/plosone/s/figures

You may also use PLOS’s free figure tool, NAAS, to help you prepare publication quality figures: https://journals.plos.org/plosone/s/figures#loc-tools-for-figure-preparation.

NAAS will assess whether your figures meet our technical requirements by comparing each figure against our figure specifications.

Attachment Submitted filename: comment for PONE-D-26-05119.docx

# Author comment: Author response to Decision Letter 1

**DOI:** 10.1371/journal.pone.0356812.r002

20 Jun 2026

Response to Reviewer and Editor Comments

Manuscript title: Adult attachment, loneliness, and self-reported sleep outcomes: A secondary analysis of the Touch Test Study

Manuscript ID: PONE-D-26-05119

We thank the reviewer and editor for their careful and constructive feedback. We have revised the manuscript substantially and have provided a marked-up version showing changes from the previous submission. The main revisions include: aligning the central claim throughout the manuscript; revising the abstract, title, Results, Discussion, Conclusion, tables, and figure legend; clarifying the ordinal regression models and diagnostics; adding false discovery rate correction across the primary interaction tests; adding a sleep-duration sensitivity analysis; improving missing-data reporting; clarifying psychometric scoring and current-sample reliability; adding preregistration, ethics, and data availability details; and auditing the reference list and citation style.

Responses to Major Comments

Major comment 1. Main result inconsistently reported across sections

Reviewer comment: The reviewer noted that the title-page abstract, manuscript abstract, Results, Discussion, Conclusion, tables, and figure legend did not present a stable central result. In particular, the previous version sometimes referred to sleep quality, whereas the significant adjusted interaction concerned sleep duration.

Response: We agree and have corrected this throughout the manuscript. The title, abstract, Results, Discussion, Conclusion, appendix tables, and figure legend now consistently state that the only significant attachment x loneliness interaction in the adjusted models was the avoidant attachment x loneliness interaction predicting sleep duration. The revised manuscript also states explicitly that the corresponding interactions were not significant for sleep quality, sleep onset, or wake after sleep onset. We removed language that blended adjusted and unadjusted findings and interpret the adjusted models as the primary analyses.

Major comment 2. Modelling of sleep duration

Reviewer comment: The reviewer noted that treating sleep duration as an ordered ordinal outcome may be problematic because very long sleep is not necessarily more favourable than shorter sleep, and requested justification or sensitivity analysis.

Response: We agree that sleep duration is not a straightforward monotonic health outcome. We retained the ordinal logistic specification because the original response options were ordered sleep-duration categories and to maintain comparability with prior literature using the PSQI sleep-duration component. However, we acknowledge that very long sleep may not reflect a more favorable sleep outcome. To address this concern, we conducted a sensitivity analysis excluding participants reporting more than nine hours of sleep per night. This group comprised only 0.7% of the sample (n = 113). The avoidant attachment × loneliness interaction showed the same pattern and remained significant in this sensitivity analysis (OR = .997, 95% CI [.995, .999], B = -.003, p = .002). We have revised the Results and Limitations sections accordingly and now interpret the sleep-duration findings more cautiously.

Major comment 3. Multiple testing and practical significance of the key interaction

Reviewer comment: The reviewer noted that two interaction terms were tested across four sleep outcomes and requested clarification of the number of primary interaction tests, multiplicity correction, and cautious interpretation of the small interaction effect.

Response: We have revised the Analysis and Results sections to state that the primary focus was on two attachment x loneliness interaction terms across four sleep outcomes, resulting in eight primary interaction tests. We applied Benjamini-Hochberg false discovery rate correction across these eight interaction tests. The avoidant attachment x loneliness interaction predicting sleep duration remained significant after correction (FDR-adjusted p = .020). We also revised the Abstract, Results, and Discussion to describe the effect as limited and small in magnitude rather than emphasising statistical significance alone. We interpret OR = .998 as a per-unit raw-score effect" so we might want to say that in the letter as it also speaks to practical signifance. For instanace, "We also note that the reported odds ratios correspond to one-unit increases in the raw-score interaction term. Because the interaction term is derived from the product of two continuous variables, a one-unit increase represents a relatively small change on the underlying scale. Accordingly, odds ratios close to 1.00 should not be interpreted as necessarily indicating an absence of association. We have not added a detailed discussion of this point to the manuscript, but note it here in response to the reviewer’s query. In the revised manuscript, we instead focus on the limited magnitude of the observed association and avoid overemphasising statistical significance. We have also specified that our analyses were hypothesis-driven and specified a priori. Pre-registration links have been added to the manuscript.

Major comment 4. Methods and Results mismatch

Reviewer comment: The reviewer noted that the previous Methods section referred to “the interaction of both attachment dimensions,” whereas the tables did not include an anxious attachment x avoidant attachment term.

Response: We have corrected the Methods section to specify the exact model estimated for each outcome. The revised model includes avoidant attachment, anxious attachment, loneliness, the avoidant attachment x loneliness interaction, the anxious attachment x loneliness interaction, age, gender, and participation end date. We also added the model formula in the Analysis section for transparency.

Major comment 5. Missing data handling and complete-case analysis

Reviewer comment: The reviewer requested clearer reporting of missing data, including a participant flow table, number excluded at each stage, reasons for exclusion, comparison of complete-case and excluded participants, and justification of complete-case analysis.

Response: We have expanded the missing-data reporting. The revised manuscript now reports the final complete-case sample sizes for each model: N = 11,914 for sleep duration, N = 11,916 for sleep quality, N = 11,908 for sleep onset, and N = 11,901 for wake after sleep onset. The Supplementary Material now includes a participant exclusion flow table showing the full Touch Test dataset, sex/gender restriction, Healthy UK analytic restriction, and complete-case samples for each sleep model. We also added a comparison of participants included versus excluded from the sleep-duration model on age, gender, and ethnicity.

Complete-case analysis is now described explicitly in the Analysis and Limitations sections. We retained a complete-case approach because this was a secondary analysis of an existing dataset, the resulting analytic samples remained large, and missingness was primarily item-specific. We acknowledge, however, that complete-case analysis may introduce bias if data are not missing completely at random, and we therefore note that future work should consider multiple imputation or other missing-data approaches where appropriate.

Major comment 6. Covariate selection and residual confounding

Reviewer comment: The reviewer noted that covariate selection appeared data-driven and asked for stronger theoretical justification and clearer acknowledgement of unavailable confounders, including depression/anxiety symptoms, relationship status, co-sleeping/cohabitation, and socioeconomic variables.

Response: We have revised the Analysis section to justify covariate selection more clearly and no longer describe covariate inclusion as being based solely on preliminary statistical associations. Age and gender are now described as retained because both are relevant to sleep and psychosocial outcomes in prior research, and participation end date was retained to account for timing effects in the Touch Test dataset and the early COVID-19 period. We also expanded the Limitations section to state that important potential confounders were unavailable or could not be modelled, including depression and anxiety symptoms, relationship status, socioeconomic variables, cohabitation, co-sleeping arrangements, and diagnosed sleep disorders. We now explicitly acknowledge the possibility of residual confounding.

Major comment 7. Preregistration reporting

Reviewer comment: The reviewer requested the exact preregistration link, date of preregistration, statement of what was preregistered, and deviations from the original plan.

Response: We have revised the Data Collection section to include the preregistration date and archived link. The manuscript now states that the study was preregistered on October 10th 2023 prior to data analysis and gives the archived preregistration link. The preregistration specified analyses of all interactions and covariates in addition to hypotheses that suggest direction and significance. We also added a statement of deviation from the preregistered analysis plan: the three-way interaction between attachment anxiety, attachment avoidance, and loneliness was omitted because the revised analysis focused on the two theoretically specified attachment x loneliness interactions.

Major comment 8. Psychometric and scoring details for measures

Reviewer comment: The reviewer requested clearer attachment scoring, possible score ranges, current-sample reliability for loneliness, and clarification that the sleep variables were adapted PSQI items rather than the full PSQI.

Response: We have revised the Measures section. For attachment, we now explain that attachment anxiety and attachment avoidance scores were calculated separately by averaging valid responses within each six-item subscale and multiplying the mean by six, producing scores from 6 to 42. We report current-sample internal consistency for attachment anxiety (alpha = .85) and attachment avoidance (alpha = .87). For loneliness, we now report that total scores ranged from 4 to 16 and that current-sample reliability was acceptable (alpha = .73). We have revised the Measures section to specify that loneliness was assessed using a 4-item version of the UCLA Loneliness Scale rather than the full 20-item measure. We now cite the psychometric evaluation supporting this. This was conducted using the Touch Test dataset (Panayiotou et al., 2022), which identified this 4-item version as a robust and reliable measure of loneliness across adult age groups. We also describe the items included in the scale, report the score range (4–16), and provide current-sample internal consistency (α = .73)."

For sleep, we clarify that the outcomes were selected items adapted from the PSQI and were analysed as separate self-reported sleep outcomes rather than as a composite PSQI score.

Major comment 9. Ordinal logistic regression diagnostics

Reviewer comment: The reviewer requested diagnostic checks for the ordinal models, including the proportional odds/parallel lines assumption, model fit statistics, thresholds/cutpoints, centering/standardisation, and multicollinearity.

Response: We have revised the Analysis and Results sections and added model diagnostics in the Supplementary Material. The revised Analysis section now states that predictors were mean-centred before interaction terms were created and that multicollinearity was assessed using variance inflation factors, all below 1.40. The Supplementary Material reports model fit statistics, Nagelkerke R-squared values, Pearson and deviance goodness-of-fit tests, and tests of the proportional odds assumption for all four ordinal logistic regression models. Because the test of parallel lines was significant for most sleep measures, the Results and Discussion now state that the findings should be interpreted cautiously. However, we retained the ordinal specification because it reflects the original ordered response categories and because sensitivity analyses yielded substantively similar conclusions.

Major comment 10. Data availability and ethics statements

Reviewer comment: The reviewer noted that the data availability and ethics statements were inconsistent with the submission metadata and requested harmonisation.

Response: We have revised both statements. The Data Availability Statement now states that the data underlying this study are available from the UK Data Service ReShare repository, identifies the dataset as The Touch Test: Cross-sectional survey data, 2020, and provides the direct access link. We removed wording implying that the dataset is available only with permission from the original author. The ethics statement now identifies the University of Bristol School of Psychological Science Research Ethics Committee, the approval date (03/11/2023), and ethics approval code 16127.

Responses to Section-by-Section Comments

Title

Reviewer comment: The reviewer suggested that the previous title was too narrow and misleading because it promised effects on sleep quality, whereas the paper examined four sleep outcomes and the key adjusted interaction concerned sleep duration.

Response: We changed the title to a neutral title reflecting the full scope of the study: “Adult attachment, loneliness, and self-reported sleep outcomes: A secondary analysis of the Touch Test Study.”

Abstract

Reviewer comment: The reviewer requested substantial revision of the abstract, including consistent sample size, final analytic N, exact significant and non-significant interaction findings, and a cautious conclusion.

Response: We rewrote the abstract. It now reports the eligible sample (N = 15,049), the final analytic sample range across the main models (N = 11,901 to N = 11,916), the only significant adjusted interaction (avoidant attachment x loneliness predicting sleep duration), and the non-significant interactions for sleep quality, sleep onset, and wake after sleep onset. The conclusion now describes the findings as limited evidence and notes that the observed moderation effect was specific to sleep duration and small in magnitude. The online submission abstract will be updated to match the manuscript abstract exactly.

Introduction

Reviewer comment: The reviewer recommended distinguishing moderation from mediation, trimming speculative REM-sleep biological discussion, clarifying preregistration, and framing the broad moderation hypothesis cautiously.

Response: We revised the Introduction to focus on psychological and behavioural pathways linking attachment, loneliness, and sleep, rather than REM-sleep biological mechanisms that were not directly tested. The speculative REM paragraph was removed. We clarified the rationale for moderation by explaining that the association between loneliness and sleep may depend on attachment-related individual differences. We also removed unsupported preregistration phrasing from the Introduction and added preregistration details in the Methods. The Discussion now explicitly states that the moderation hypothesis was not supported across most sleep outcomes.

Methods: Data collection

Reviewer comment: The reviewer requested clarity on the preregistered secondary analysis, date of analysis plan, and dataset access.

Response: We revised the Data Collection section to state that the current study was a secondary analysis of the Touch Test dataset, that the data were accessed on 16/10/2023, and that the study was preregistered on October 10th 2023 before data analysis. We also identify the blocks from the original questionnaire used in the secondary analysis: loneliness, attachment style, demographics, and sleep.

Methods: Study participants

Reviewer comment: The reviewer asked for clearer justification of the analytic restrictions, including UK participants, men/women only, and no psychological or physical health condition.

Response: We revised the Study Participants section to frame the UK and gender restrictions as analytic restrictions to maximise sample size and stable model estimation. The manuscript now explains that non-UK participants and participants selecting gender as “other” were excluded because representation from these groups was substantially lower, resulting in uns

Attachment Submitted filename: Response_to_Reviewers (3).docx

# Peer review: Decision Letter 1

**DOI:** 10.1371/journal.pone.0356812.r003

**Authors:** Runtang Meng

10 Aug 2026

Adult attachment, loneliness, and self-reported sleep outcomes: A secondary analysis of the Touch Test Study

PONE-D-26-05119R1

Dear Dr. Samii,

We’re pleased to inform you that your manuscript has been judged scientifically suitable for publication and will be formally accepted for publication once it meets all outstanding technical requirements.

Within one week, you’ll receive an e-mail detailing the required amendments. When these have been addressed, you’ll receive a formal acceptance letter and your manuscript will be scheduled for publication.

An invoice will be generated when your article is formally accepted. Please note, if your institution has a publishing partnership with PLOS and your article meets the relevant criteria, all or part of your publication costs will be covered. Please make sure your user information is up-to-date by logging into Editorial Manager at Editorial Manager® and clicking the ‘Update My Information' link at the top of the page. For questions related to billing, please contact billing support.

If your institution or institutions have a press office, please notify them about your upcoming paper to help maximize its impact. If they’ll be preparing press materials, please inform our press team as soon as possible -- no later than 48 hours after receiving the formal acceptance. Your manuscript will remain under strict press embargo until 2 pm Eastern Time on the date of publication. For more information, please contact onepress@plos.org.

Kind regards,

Runtang Meng, PhD

Academic Editor

PLOS One

Additional Editor Comments (optional):

Reviewers' comments:

Reviewer's Responses to Questions

**Comments to the Author**

Reviewer #1: All comments have been addressed

Reviewer #3: All comments have been addressed

**********

2. Is the manuscript technically sound, and do the data support the conclusions?

Reviewer #1: Yes

Reviewer #3: Yes

**********

3. Has the statistical analysis been performed appropriately and rigorously?

Reviewer #1: Yes

Reviewer #3: Yes

**********

4. Have the authors made all data underlying the findings in their manuscript fully available?

The PLOS Data policy

Reviewer #1: Yes

Reviewer #3: Yes

**********

5. Is the manuscript presented in an intelligible fashion and written in standard English?

Reviewer #1: Yes

Reviewer #3: Yes

**********

Reviewer #1: (No Response)

Reviewer #3: The authors have carefully addressed the comments made. The provided version of the manuscript, which reflects the changes from the previous version, is publishable as it meets the journal's rigorous standards.

**********

what does this mean?). If published, this will include your full peer review and any attached files.

If you choose “no”, your identity will remain anonymous but your review may still be made public.

**Do you want your identity to be public for this peer review?**  For information about this choice, including consent withdrawal, please see our Privacy Policy

Reviewer #1: No

Reviewer #3: **Yes:**  Francisco Paz Rodríguez

**********

# Editor report: Acceptance letter

**DOI:** 10.1371/journal.pone.0356812.r004

**Authors:** Runtang Meng

PONE-D-26-05119R1

PLOS One

Dear Dr. Samii,

I'm pleased to inform you that your manuscript has been deemed suitable for publication in PLOS One. Congratulations! Your manuscript is now being handed over to our production team.

At this stage, our production department will prepare your paper for publication. This includes ensuring the following:

* All references, tables, and figures are properly cited

* All relevant supporting information is included in the manuscript submission,

* There are no issues that prevent the paper from being properly typeset

You will receive further instructions from the production team, including instructions on how to review your proof when it is ready. Please keep in mind that we are working through a large volume of accepted articles, so please give us a few days to review your paper and let you know the next and final steps.

Lastly, if your institution or institutions have a press office, please let them know about your upcoming paper now to help maximize its impact. If they'll be preparing press materials, please inform our press team within the next 48 hours. Your manuscript will remain under strict press embargo until 2 pm Eastern Time on the date of publication. For more information, please contact onepress@plos.org.

You will receive an invoice from PLOS for your publication fee after your manuscript has reached the completed accept phase. If you receive an email requesting payment before acceptance or for any other service, this may be a phishing scheme. Learn how to identify phishing emails and protect your accounts at https://explore.plos.org/phishing.

If we can help with anything else, please email us at customercare@plos.org.

Thank you for submitting your work to PLOS One and supporting open access.

Kind regards,

PLOS One Editorial Office Staff

on behalf of

Dr. Runtang Meng

Academic Editor

PLOS One
